# Trans-NanoSim characterizes and simulates nanopore RNA-sequencing data

**DOI:** 10.1093/gigascience/giaa061

**Published:** 2020-06-10

**Authors:** Saber Hafezqorani, Chen Yang, Theodora Lo, Ka Ming Nip, René L Warren, Inanc Birol

**Affiliations:** Canada's Michael Smith Genome Sciences Centre, 100 - 570 W 7th Ave, Vancouver, BC Cancer, BC V5Z 4S6 Canada; Bioinformatics Graduate Program, University of British Columbia, 100 - 570 W 7th Ave, Vancouver, BC Cancer, BC V5Z 4S6 Canada; Canada's Michael Smith Genome Sciences Centre, 100 - 570 W 7th Ave, Vancouver, BC Cancer, BC V5Z 4S6 Canada; Bioinformatics Graduate Program, University of British Columbia, 100 - 570 W 7th Ave, Vancouver, BC Cancer, BC V5Z 4S6 Canada; Canada's Michael Smith Genome Sciences Centre, 100 - 570 W 7th Ave, Vancouver, BC Cancer, BC V5Z 4S6 Canada; Canada's Michael Smith Genome Sciences Centre, 100 - 570 W 7th Ave, Vancouver, BC Cancer, BC V5Z 4S6 Canada; Bioinformatics Graduate Program, University of British Columbia, 100 - 570 W 7th Ave, Vancouver, BC Cancer, BC V5Z 4S6 Canada; Canada's Michael Smith Genome Sciences Centre, 100 - 570 W 7th Ave, Vancouver, BC Cancer, BC V5Z 4S6 Canada; Canada's Michael Smith Genome Sciences Centre, 100 - 570 W 7th Ave, Vancouver, BC Cancer, BC V5Z 4S6 Canada; Department of Medical Genetics, University of British Columbia, 2350 Health Science Mall, Vancouver, BC V6T 1Z3, Canada

**Keywords:** nanopore sequencing, sequence simulation, transcriptome, RNA-seq

## Abstract

**Background:**

Compared with second-generation sequencing technologies, third-generation single-molecule RNA sequencing has unprecedented advantages; the long reads it generates facilitate isoform-level transcript characterization. In particular, the Oxford Nanopore Technology sequencing platforms have become more popular in recent years owing to their relatively high affordability and portability compared with other third-generation sequencing technologies. To aid the development of analytical tools that leverage the power of this technology, simulated data provide a cost-effective solution with ground truth. However, a nanopore sequence simulator targeting transcriptomic data is not available yet.

**Findings:**

We introduce Trans-NanoSim, a tool that simulates reads with technical and transcriptome-specific features learnt from nanopore RNA-sequncing data. We comprehensively benchmarked Trans-NanoSim on direct RNA and complementary DNA datasets describing human and mouse transcriptomes. Through comparison against other nanopore read simulators, we show the unique advantage and robustness of Trans-NanoSim in capturing the characteristics of nanopore complementary DNA and direct RNA reads.

**Conclusions:**

As a cost-effective alternative to sequencing real transcriptomes, Trans-NanoSim will facilitate the rapid development of analytical tools for nanopore RNA-sequencing data. Trans-NanoSim and its pre-trained models are freely accessible at https://github.com/bcgsc/NanoSim.

## Background

RNA sequencing (RNA-seq) is a cornerstone technology that has helped further our understanding of transcriptomes [1]. Third-generation single-molecule sequencing technologies such as those from Oxford Nanopore Technologies (ONT, Oxford, UK) are proving invaluable for isoform-level analyses. For example, ONT reads 1–100 kb in length permit identification and quantification of most full-length isoforms in the human transcriptome and enable various complex feature analyses [2–5]. In recent years, there has been an increase in the development of novel algorithms to leverage the power of this technology, including *de novo* assembly, alignment and mapping, and structural variant detection [6–12]. In this active field of research, simulated data with a known ground truth provide a cost-effective means to help develop, refine, and benchmark these tools.

Long-read simulators have been developed for ONT genomic reads [13, 14]. DeepSimulator [14] uses a context-dependent deep learning model to simulate the electrical current signals, which are decoded into sequence reads using any off-the-shelf base-calling method. Although it may facilitate the development of base-calling algorithms, DeepSimulator cannot provide the ground truth at the base level. On the other hand, as a base-level simulator, NanoSim [13] first utilizes statistical models to learn the characteristics of sequencing libraries and then applies those models to simulate ONT genomic reads directly. Although proven to have advanced the development of various bioinformatics analysis tools, NanoSim's initial development was centered on simulating genomic reads [12, 15]. Neither of these tools is specifically designed to capture and reproduce transcriptome-specific features such as transcript expression profiles and intron retention (IR) events. While transcript expression levels inform the biological state of a transcriptome, IR, as one of the main forms of alternative splicing, contributes to the functional complexity of eukaryotic transcriptomes [16]. ONT reads have the potential to capture complex IR events involving multiple introns, thus allowing researchers to investigate IR at isoform-level resolution. In addition, the inadequacy of base callers to detect timespan in the signal data often results in homopolymer expansion and contraction events, represented by significantly higher deletion rates in homopolymer regions. Despite these homopolymer errors accounting for many, if not the majority, of the errors in ONT reads, no ONT read simulator can accurately simulate them. Taking all these into consideration, there is currently an unmet need for an ONT RNA-seq simulator, which can aid the development of transcriptome analysis methods without the expense of sequencing experiments.

## Findings

Here we present further developments of NanoSim and introduce Trans-NanoSim, which is specifically designed for the ONT transcriptome sequencing platform. This versatile tool mimics the technical features of nanopore RNA-Seq data including read error modes, read length distribution, and homopolymer artefacts, which might be affected by different library preparation methods and base-calling algorithms. Furthermore, Trans-NanoSim can be trained to characterize transcriptome-specific features such as expression patterns and IR events for more accurate simulation. To demonstrate the performance of Trans-NanoSim, we chose 3 sets of publicly available experimental ONT reads for training and simulation, including human NA12878 direct RNA, complementary DNA (cDNA) 1D^2^, and mouse cDNA 1D libraries (Supplementary Note 1). Through benchmarking the similarity between experimental and simulated reads, we show that Trans-NanoSim consistently outperforms the genomic simulator DeepSimulator, on all 3 datasets.

**Figure 1:**
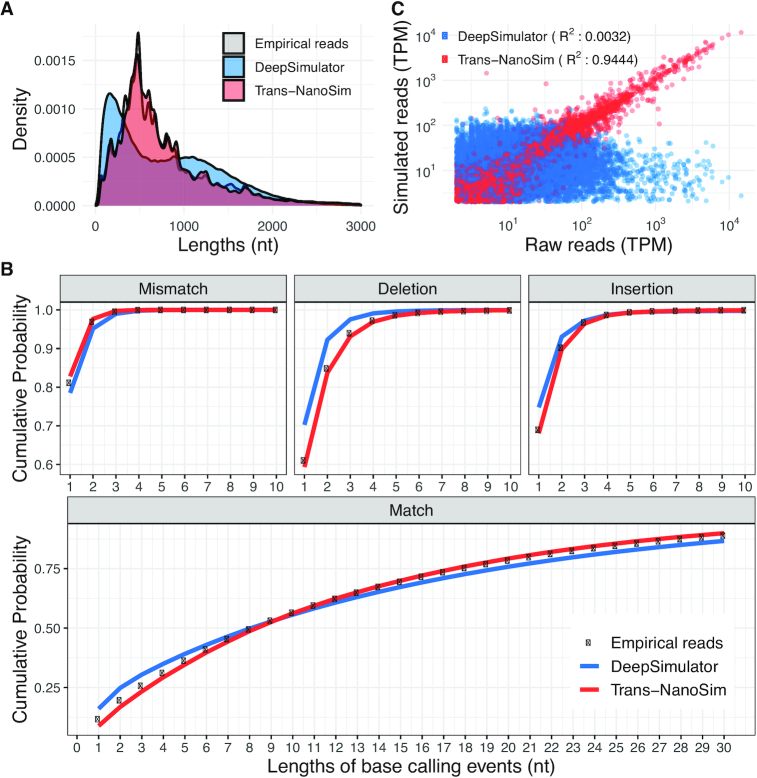
Benchmarking Trans-NanoSim and DeepSimulator on the human direct RNA dataset. **A**. Comparison of length distributions of experimental reads and simulated reads generated by Trans-NanoSim and DeepSimulator. **B**. The length of consecutive match/error bases of empirical and simulated reads, as indicated. **C**. Transcript expression levels measured from simulated reads versus the same measured from experimental reads.

Unlike short reads generated from second-generation sequencing technologies, ONT reads have very long and non-uniform lengths. Thus, read length is a key feature to preserve in simulation. The read length distribution of transcriptomic data is jointly influenced by sequencing techniques, sample preparation protocols (often leading to reads derived from partial transcripts), and transcriptomic variables, such as transcript lengths and expression levels (for the latter, different expression profiles may result in different read length distributions). Therefore, to capture this relationship between expression levels and read lengths, we profiled 3 datasets and then simulated reads with Trans-NanoSim and DeepSimulator (Supplementary Note 2). For the human direct RNA dataset, the length distribution of simulated reads generated by Trans-NanoSim (mean [SD] = 807 [0.75] nucleotides [nt] determined by ordinary nonparametric bootstrapping 1,000 times using the boot command in R, Fig. S1) followed the empirical read length distribution (mean = 815 nt) closely (Fig. 1A). Although we configured DeepSimulator to preserve the mean read length of empirical reads (mean = 808 nt), DeepSimulator still generated a bimodal length distribution with a mode of ∼150 nt. We suspect that this limitation is due to the predefined read-length distributions of DeepSimulator, while the ONT read length cannot be simply described by a single statistical distribution, as elucidated by previous studies [13]. Furthermore, DeepSimulator, being a genomic read simulator, does not associate the isoform expression levels with read lengths.

Next, we aligned the simulated and empirical reads to the reference genome and evaluated the length of consecutive match/error bases in both sets (Supplementary Note 2). While the error rate of the empirical reads from the human direct RNA dataset was 10.53%, the simulated reads generated by Trans-NanoSim and DeepSimulator were 10.44% and 11.09%, respectively (Supplementary Table S1). Combined with the length distribution of base-calling events, it is evident that Trans-NanoSim mimics error and match events more closely to the experimental data (Fig. 1B).

For a transcriptome sequence simulator, it is critical to output the correct number of simulated reads for each transcript (i.e., amount that reflects the expected expression level of a given transcript). To evaluate whether a simulated dataset generated by both tools accounts for transcript isoform usage and expression level, we used the "quantify" module in Trans-NanoSim to compute the transcript expression levels with both empirical and simulated reads (Supplementary Note 2). The coefficient of determination (*R*^2^) between the estimated transcript abundance of the empirical human direct RNA dataset and the simulated dataset generated by Trans-NanoSim is 0.9444, indicating that the observed raw transcript expression level is well replicated by Trans-NanoSim (Fig. 1C). In contrast, the *R*^2^ value for DeepSimulator simulated reads is 0.0032, which suggests that the transcript abundance in the simulated dataset is independent of its counterpart in the empirical one. Because genomic simulators do not require expression profiles as input, it is expected that this desirable feature is missing.

To the best of our knowledge, Trans-NanoSim is the first transcriptome sequence simulator that provides IR modelling. Considering the human direct RNA dataset as an example, the IR modelling module of Trans-NanoSim identified 2,872 transcripts with ≥1 retained intron, and nearly half of them (1,285 transcripts) were expressed at >2 transcripts per million (TPM). Interestingly, we identified as many as 6 retained introns in 1 highly expressed transcript (Ensembl transcript ID: ENST00000425660, TPM = 1,433). The IR modelling module also reports the transitional probability of each intron being retained based on the state of the previous intron, a model that the pipeline uses for read simulations. In the human direct RNA dataset, only 0.41% of reads spanned the first intron of the represented transcript. However, given that an intron is retained, the probability of observing the subsequent intron being retained increased to 17.12%.

**Figure 2:**
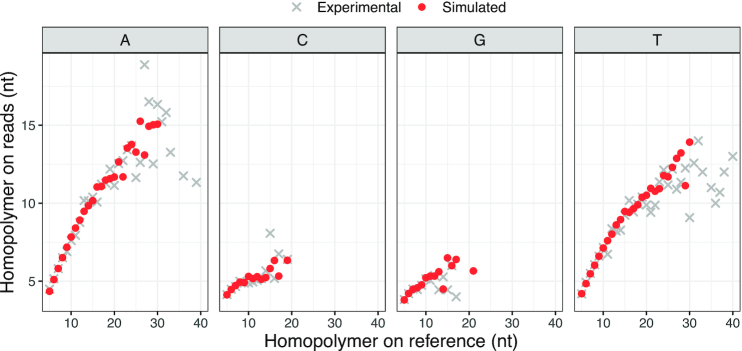
Homopolymer simulation performance on the human direct RNA dataset. The *x*-axis shows the reference homopolymer length (nt) and *y*-axis is the mean homopolymer length (nt) on corresponding reads. The distributions for A and T homopolymers are trimmed at 40 nt.

Another novel feature that we introduce to Trans-NanoSim is homopolymer length modelling, which applies to both genome and transcriptome simulations. It is known that the high error rate of ONT reads is partially due to the base-calling artefact in homopolymer regions [17] and the base-calling errors, mainly deletions, in those regions are substantially higher than in non-homopolymer regions (Supplementary Table S2). Trans-NanoSim simulates homopolymer of each base type individually, and in our experiments, the mean homopolymer length is largely consistent between simulated and experimental reads (Fig. 2). Our analysis revealed a linear correlation between the homopolymer length on the reference compared to the sequencing reads. However, as the homopolymer length increases, fewer data points were observed, thus widening the confidence interval. As a result, we observed a larger variation between simulated length and experimental lengths for A and T homopolymers longer than 20 nt and C and G homopolymers longer than 15 nt. We note that in the experimental long-read datasets used herein, at most only 0.08% and <0.01% of reads containing these homopolymer lengths were observed, respectively, and will likely represent rare occurrences in ONT data.

Finally, we evaluated the computational performance of Trans-NanoSim and DeepSimulator through characterizing and simulating 687,192 reads describing the human reference transcriptome (Supplementary Note 2). Although both tools allow users to train a custom model with any dataset, the authors of DeepSimulator noted that this step is computationally intensive and advised their users against trying it [18]. In contrast, in a typical run, it takes Trans-NanoSim <1 h to train and an additional few minutes to compute the expression profile with 4 processors. In the simulation stage, Trans-NanoSim ran for 2h11m with peak memory of 526 MB, while DeepSimulator ran for 1d8h32m in total (with 5h46m to simulate signals and 1d2h46m for base calling) with peak memory of 17.22 GB (Supplementary Tables S3 and S4). Trans-NanoSim also supports multi-processing, which reduces the runtime significantly, but at the cost of increased memory usage (Supplementary Fig. S2, Table S5). The runtime of Trans-NanoSim is proportional to the number of reads to be simulated, with a fixed time usage for reading in profiles. The effect of multiprocessing starts to saturate with 12 CPUs when processing <60,000 reads, while with more reads, this saturation point is observed with a greater number of CPUs. Even with only 4 processors, there is a substantial reduction in runtime (∼75% less than the same run on a single CPU), which took 33 minutes to simulate 687,192 human direct RNA reads.

We recapitulated our results by repeating all the analyses presented here on human cDNA 1D^2^ and mouse cDNA sequencing data and obtained similar findings (Supplementary Figs S3 and S4, respectively, and Table S1). We noticed that even though the error rates in the raw reads can vary from experiment to experiment, DeepSimulator always generates reads with similar error rates and length distribution, while Trans-NanoSim can adapt to different sequencing libraries and simulates base-calling events that are true to the platform.

In this work, we report on results from comprehensive benchmarking experiments to illustrate Trans-NanoSim's performance on 3 ONT RNA-seq datasets with different sequencing data types: direct RNA, cDNA 1D^2^, and cDNA 1D. Our evaluations demonstrate the robustness of Trans-NanoSim in learning and mimicking the length distribution, sequence error profiles, and homopolymer runs of nanopore RNA-seq reads. Moreover, Trans-NanoSim provides a solution to the characterization of transcriptome-specific features, such as isoform expression and IR events, which cannot be addressed by genomic read simulators. As a fast and memory-efficient ONT read simulator, Trans-NanoSim is feasible to run on a standard modern-day laptop computer. We anticipate that it will offer an important functionality to the community and it will facilitate the development of various base-level bioinformatics algorithms that leverage the potential of long nanopore reads, including transcriptome assembly, alignment and quantification, structural variant detection, and novel isoform identification.

## Methods

### Trans-NanoSim workflow overview

**Figure 3:**
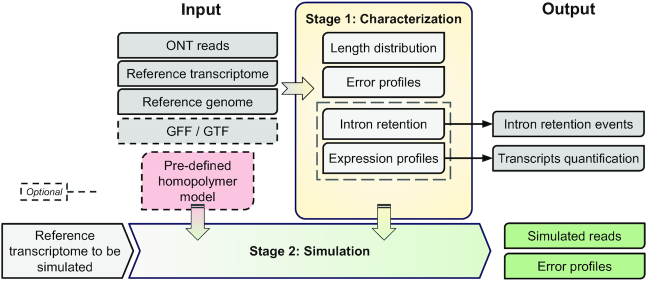
Schematic overview of the Trans-NanoSim pipeline. The first stage (Characterization) of the pipeline aligns input ONT transcriptome reads against the reference transcriptome and genome to statistically model the read length distribution and error modes. It also optionally detects intron retention events and quantifies transcript expression. These profiles alongside the homopolymer model are then used in the second stage (Simulation) to generate simulated reads, also reporting their associated error profiles.

The workflow of Trans-NanoSim consists of 2 stages: characterization of experimental reads and simulation from a reference transcriptome (Fig. 3). In the characterization stage, experimental reads are aligned against the reference transcriptome to infer their source transcript, which is essential for read length analysis and transcript expression quantification. Reads are also aligned against the reference genome to compute statistical models for read error modes. Both genomic and transcriptomic alignments are used to model intron retention events. We also provide pre-trained models along with this work for users to use directly without training. Next, according to these models, reads are simulated given a reference transcriptome and genome. For each read to be simulated, the source reference transcript is selected on the basis of the expression profile. Then, a sequence is extracted from that transcript according to the length distribution model, and it is modified with respect to the IR and error models.

### Length distribution characterization and simulation

Previous versions of NanoSim used an empirical cumulative density function to simulate the length distribution of reads. In the current version of the pipeline, NanoSim uses kernel density estimation (KDE), which captures underlying patterns in the read length distributions, and avoids overfitting. We also replace the binning strategy in simulating the alignment ratio on each read with KDE, resulting in a smoother simulated read length distribution. Theoretically, nanopore transcriptome sequencing can yield reads of the same length as the original messenger RNA molecule. However, in practice, ONT reads are often shorter than their corresponding mesenger RNA molecules owing to experimental or data acquisition artefacts, and thus they may represent partial transcripts. Therefore, it is crucial to consider the length of the reference transcript when simulating the length distribution of simulated ONT reads. To achieve this, we use a 2D KDE model and measure the length of an ONT read relative to the length of the source transcript. Furthermore, unaligned regions on both ends of each read are also subjected to length distribution analysis. We follow the same KDE model approach as described to model their length distributions separately.

We note that the percentage of antisense sequences in cDNA and direct RNA sequences may be substantially different. To capture this information, Trans-NanoSim automatically infers the strand ratio by calculating the percentage of reads that are in the same direction as the annotated strand. This strand ratio is then used to assign the orientation of reads accordingly during the simulation stage.

### Intron retention characterization and simulation

Trans-NanoSim is able to detect and model IR events for ONT transcriptome reads. Based on alignments to intronic regions, it uses a Markov chain model to calculate the transitional probabilities between the states of spliced and retained introns, given the state of the previous intron. This feature is not part of the characterization phase by default. To enable this option, a transcript annotation file in GTF/GFF format needs to be provided. This functionality can also be invoked in a stand-alone module (detect_ir), enabling users to only detect and model IR events without characterizing or simulating reads. The module outputs comprehensive information on the location of the detected IR events based on input ONT reads.

### Transcript abundance quantification and simulation

We have incorporated a pipeline [19] to estimate transcript abundance based on reference transcriptome alignments (J. Simpson, personal communication). The pipeline relies on minimap2 [7] with -p0 flag to retain all secondary mappings and then utilizes an expectation maximization approach similar to RSEM [20] to assign multi-mapping reads. It is a stand-alone module (quantify) that outputs transcript abundance in TPM values, which can be used in the simulation stage. Users may also provide their own expression profile in tab-delimited format, describing empirical or theoretical distributions, if preferred. During simulation, these transcript abundance values are used to calculate the probability of an isoform being selected and ultimately the number of constituent reads of each isoform.

### Error mode characterization and simulation

Statistical modelling of error patterns in long nanopore reads was proven to be effective in mimicking the sequencing platform [13]. In Trans-NanoSim, we build on the same mixture models to deal with transcriptome reads as these patterns are shared among different library preparation methods and datasets. According to the alignments, reads are classified into 2 groups: aligned and unaligned. For each group, we consider specific characterization and modelling approaches. As for the aligned reads, we consider their aligned bases for further error rate analysis. The lengths of indels and mismatches are drawn from Weibull/Geometric and Poisson/Geometric mixture models, respectively. We also calculate the transitional probability between every 2 consecutive base call errors using a Markov chain model. We reimplemented the model-fitting function of NanoSim in Python (formerly in R) and allowed multi-threading to expedite the fitting process. Unaligned reads may provide crucial information about the nature of ONT sequencing experiments, and thus we chose to model the length distribution of the unaligned reads as well. For this purpose, we extract sequences from reference transcripts based on their length distribution and apply an arbitrarily high error rate (default, 90%). However, because it is impossible to trace their source transcript molecule, unaligned reads are not included in the error rate analysis.

### Homopolymer characterization and simulation

Previous versions of NanoSim have a *k*-mer bias parameter (-k-mer) in the simulation stage that effectively compresses all homopolymers longer than *n* into *n*-mers. However, it does not simulate homopolymer expansion events nor is it an accurate representation of the distribution of read homopolymer lengths. In our analysis and the datasets inspected, we observed that the homopolymer length on sequencing reads is consistent with a normal distribution. Furthermore, the mean and associated standard deviation of homopolymer lengths on those same reads is linearly proportional to the reference homopolymer length (Supplementary Fig. S5). In the simulation stage, Trans-NanoSim first finds homopolymers >*n* in the sequence extracted from the reference. Given the reference homopolymer length, the mean and standard deviation, which are used to generate the normal distribution, are calculated from segmented and linear regression models, respectively. The homopolymer length to be simulated is then drawn from the constructed normal distribution, and the extracted sequence is modified accordingly. Depending on the base caller used and sequencing types, the distribution of read homopolymer lengths can vary; thus, we provide pre-trained models to simulate genome and transcriptome reads base called with Albacore, Guppy's default model, and Guppy's flip-flop model.

## Availability of Supporting Source Code and Requirements

Trans-NanoSim is developed in Python. Source code and pre-trained models for this work are freely accessible at https://github.com/bcgsc/NanoSim (Licence: GPL-3). Trans-NanoSim is also registered in the bio.tools (biotools: Trans-NanoSim) and SciCrunch (RRID:SCR_018243) databases.

## Availability of Supporting Data and Materials

Snapshots of our code and other supporting data are openly available in the *GigaScience* repository, GigaDB [21].

## Additional Files

Supplementary Figure S1 - Bootstrapping results for length distribution analyses

Supplementary Figure S2 - Runtimes for multiprocessing

Supplementary Figure S3 - Benchmarking Trans-NanoSim and DeepSimulator on the human cDNA 1D2 dataset

Supplementary Figure S4 - Benchmarking Trans-NanoSim and DeepSimulator on the mouse cDNA dataset

Supplementary Figure S5 - Homopolymer characterization of human NA12878 direct RNA dataset

Supplementary Table S1 - Error rates in empirical and simulated reads

Supplementary Table S2 - Error rates in homopolymer regions and non-homopolymer regions for human direct RNA dataset

Supplementary Table S3 - Runtime usage in simulating Human direct RNA dataset for Trans-NanoSim and DeepSimulator

Supplementary Table S4 - Memory usage (maximum resident set size in GB) in simulating Human direct RNA dataset for Trans-NanoSim and DeepSimulator

Supplementary Table S5 - Trans-NanoSim multiprocessing memory usage (maximum resident set size in GB) with IR modelling

Supplementary Note 1 - Datasets

Supplementary Note 2 - Simulating reads from human and mouse reference transcriptomes and analyses

giaa061_GIGA-D-20-00043_Original_Submission

giaa061_GIGA-D-20-00043_Revision_1

giaa061_Response_to_Reviewer_Comments_Original_Submission

giaa061_Reviewer_1_Report_Original_Submission Camille Marchet -- 3/10/2020 Reviewed

giaa061_Reviewer_2_Report_Original_Submission Andrey D. Prjibelski, M.Sc. -- 3/13/2020 Reviewed

giaa061_Reviewer_2_Report_Revision_1 Andrey D. Prjibelski, M.Sc. -- 4/22/2020 Reviewed

giaa061_Supplemental_File

## Abbreviations

cDNA: complementary DNA; CPU: central processing unit; IR: intron retention; kb: kilobase pairs; KDE: kernel density estimation; nt: nucleotides; ONT: Oxford Nanopore Technologies; RNA-seq: RNA sequencing; SD: standard deviation; TPM: transcripts per million.

## Competing Interests

The authors declare that they have no competing interests.

## Funding

This work was supported by Genome Canada and Genome BC(281ANV); Genome Canada, Genome BC, Genome Quebec, and Genome Alberta (243FOR); and by the National Human Genome Research Institute of the National Institutes of Health (R01HG007182). Scholarship funding was provided by the University of British Columbia and the Natural Sciences and Engineering Research Council of Canada. The content reported is solely the responsibility of the authors and does not necessarily represent the official views of the funding organizations.

## Authors' Contributions

S.H. and C.Y. contributed equally to this work. I.B., S.H., and C.Y. conceived and designed the study. S.H. and C.Y. implemented the algorithm with the help of T.L., K.M.N., and R.L.W. S.H. drafted and all the other authors reviewed, edited, and approved the final manuscript.
