# Supplementary material for: Trans-NanoSim characterizes and simulates nanopore RNA-sequencing data
Source: Gigascience. 2020 Jun 10;9(6):giaa061. doi: 10.1093/gigascience/giaa061 (PMC7285873; doi:10.1093/gigascience/giaa061)
Supplement: giaa061_GIGA-D-20-00043_Revision_1 [file giaa061_giga-d-20-00043_revision_1.pdf]

GigaScience

# Trans-NanoSim characterizes and simulates nanopore RNA-seq data

--Manuscript Draft--

| | |
|---|---|
| **Manuscript Number:** | GIGA-D-20-00043R1 |
| **Full Title:** | Trans-NanoSim characterizes and simulates nanopore RNA-seq data |
| **Article Type:** | Technical Note |
| **Funding Information:** | Genome Canada (281ANV) — Dr. Inanc Birol<br>Genome Canada (243FOR) — Dr. Inanc Birol<br>National Human Genome Research Institute (R01HG007182) — Dr. Inanc Birol<br>Canadian Network for Research and Innovation in Machining Technology, Natural Sciences and Engineering Research Council of Canada — Dr. Inanc Birol |
| **Abstract:** | Background: Compared to second-generation sequencing technologies, third-generation single-molecule RNA sequencing has unprecedented advantages; the long reads it generates facilitate isoform-level transcript characterization. In particular, the Oxford Nanopore Technology sequencing platforms have become more popular in recent years due to their relatively high affordability and portability compared to other third-generation sequencing technologies. To aid the development of analytical tools that leverage the power of this technology, simulated data provides a cost-effective solution with ground truth. However, nanopore sequence simulator targeting transcriptomic data is not available yet.<br>Findings: We introduce Trans-NanoSim, a tool that simulates reads with technical and transcriptome- specific features learnt from nanopore RNA-seq data. We comprehensively benchmarked Trans-NanoSim on direct RNA and cDNA datasets describing human and mouse transcriptomes. Through comparison against other nanopore read simulators, we show the unique advantage and robustness of Trans-NanoSim in capturing the characteristics of nanopore cDNA and direct RNA reads.<br>Conclusions: As a cost-effective alternative to sequencing real transcriptomes, Trans-NanoSim will facilitate the rapid development of analytical tools for nanopore RNA-seq data. Trans-NanoSim and its pre- trained models are freely accessible at https://github.com/bcgsc/NanoSim . |
| **Corresponding Author:** | Inanc Birol<br>British Columbia Cancer Agency<br>Vancouver, CANADA |
| **Corresponding Author Secondary Information:** | |
| **Corresponding Author's Institution:** | British Columbia Cancer Agency |
| **Corresponding Author's Secondary Institution:** | |
| **First Author:** | Saber Hafezqorani |
| **First Author Secondary Information:** | |
| **Order of Authors:** | Saber Hafezqorani<br>Chen Yang<br>Theodora Lo<br>Ka Ming Nip<br>René L Warren<br>Inanc Birol |
| **Order of Authors Secondary Information:** | |
| **Response to Reviewers:** | [A formatted version of our Response to Reviewers is attached as our cover letter] |

Powered by Editorial Manager® and ProduXion Manager® from Aries Systems Corporation

Dear Dr. Nogoy,
Thank you for your consideration of our manuscript. We appreciate the thorough and constructive comments from you and our reviewers. In our revised submission, we have addressed the concerns raised, as outlined below, and edited the manuscript accordingly. We also registered Trans-NanoSim in bio.tools and SciCrunch databases and included the identifiers in the manuscript as requested.
Sincerely,
Saber Hafezqorani, Chen Yang, and Inanc Birol
Canada’s Michael Smith Genome Sciences Centre
British Columbia Cancer Agency

REVIEWER 1
1. "The alignment experimental reads against the reference transcriptome and genome" I would like to clarify whether this order is 1/ transcriptome then 2/ genome here?
Response: We align each read set to both reference transcriptome and to reference genome. The genome alignments are used to detect retained introns, so we can compute the error rates more accurately. The transcriptome alignments are used to assign the source transcript for each read, which is essential for read length distribution analysis and transcript expression level quantification. Both genome and transcriptome alignments are required to model intron retention events. In other words, the alignment order has no effect on the characterization phase. We have revised the text in the 1st paragraph in Methods to clarify this issue.
2. A "staircase effect" can be observed because of truncated reads in ONT experiment. I could not see a mention to truncated reads nor to this effect in the article, however I think this can be an important one. Can this be modeled within the length distribution?
Response: As noted, nanopore reads are often shorter than their corresponding mRNA molecules due to experimental or data acquisition artefacts, and thus they may represent partial transcripts. In our revised manuscript, we further clarified and explained how we consider this in our analysis.
Trans-NanoSim models the length distribution of nanopore RNA-seq reads based on the primary alignment of these reads to the reference transcriptome. During simulation, a transcript is selected from the expression profiles and read lengths are then selected from the read length distribution models to generate synthetic reads from different parts of that given transcript. We modified the following sections of our manuscript to clarify this issue:
• 4th paragraph of Findings section: We mention several reasons that cause nanopore reads to be often shorter than their corresponding mRNA molecules.
• “Length distribution characterization and simulation” - Methods section: We clarify that reads with varying lengths may be derived from different parts of a given transcript.
3. The unaligned bases are not taken into account to estimate the error rate (though the unaligned reads are used to determine the length distribution). This is a (reasonable) choice, which limitations should be clearly discussed.
Response: To calculate the error rate or accuracy for ONT reads in Trans-NanoSim, the aligned length of query sequence is used as the denominator. We note that, for this definition we are following the convention set in other studies [1, 2]. Our justification is, since the source transcript molecule is unknown for unaligned reads, it is not possible to include unaligned reads for error rate estimation. The limitation of this formula is that the error rate could be slightly inflated. We added a sentence at the 6th paragraph of “Method” section to clarify this.
4. "It is observed that the homopolymer lengths on reads follow normal distribution [...]". Do you have other sources you could cite about this?
Response: This statement is based on our own analysis, and we edited the “Homopolymer characterization and simulation” paragraph to clarify it. We also describe this analysis at 8th paragraph of “Findings” section (homopolymer modeling). The following sentences are from that paragraph:
“Trans-NanoSim simulates homopolymer of each base type individually, and in our experiments, the mean homopolymer length is largely consistent between simulated and experimental reads (Figure 2). Our analysis revealed a linear correlation between the homopolymer length on the reference compared to the sequencing reads.”
5. I think sometimes the text contains too many repetitions. For instance, " In this work, we introduce the first ONT transcriptome sequence simulator [...]" could be removed as this has already been clearly stated before.
Response: Thanks for the constructive comment to improve the readability of the text. In this revision, we thoroughly reviewed the manuscript to increase its readability and removed the repetitive phrases.
REVIEWER 2
Major comment:

Powered by Editorial Manager® and ProduXion Manager® from Aries Systems Corporation

1. My main suggestion is to add a comparison against IsoSeqSim (https://github.com/yunhaowang/IsoSeqSim). Although it was released quite some time ago and may not have such functionality as introducing intron retention, it still has main simulation features such as truncating transcripts and introducing errors according to given profiles.
Response: We thank the reviewer for bringing this tool to our attention. After careful thought and in communication with our editor we decided not to include benchmarks against IsoSeqSim. While we do not have any reason to doubt the validity of the approach implemented in IsoSeqSim, we note that it neither has a peer-reviewed publication nor a preprint describing the work. Further, we cannot determine its usage in other studies, hence its impact in the field. At the time of this writing (March 27, 2020), a Google search for “IsoSeqSim” returned only four hits, all of which were pages created by the maintainer of IsoSeqSim. According to the method’s GitHub page (https://github.com/yunhaowang/IsoSeqSim), the repository has not been updated for at least two years. We also do not see any commits, forks, pull-requests, stars, or issues (opened or closed) from other users. Thus, we conclude that IsoSeqSim is not a mature enough tool to count as being part of the state-of-the-art.
Minor comments:
1. Would be useful to provide some trained models for 1-2 typical ONT experiments in the package to allow a user to make a quick start.
Response: We totally agree that providing pre-trained models would be beneficial for users to make a quick start. In this regard, we provide several models for users to use directly without training. We mention this throughout the manuscript:
• Conclusion section in “Abstract”
• Trans-NanoSim workflow overview section in “Methods”
• Availability of supporting source code and requirements section

2. For the same reason, it could be helpful to add the possibility to simulate reads without transcript abundance file. Instead of real abundances, one can use some approximation (e.g. negative binomial distribution).
Response: We thank the reviewer for this suggestion. We agree that the transcript expression levels change between different experiments, and users may want to test different scenarios. We note that, Trans-NanoSim is quite flexible in this regard; it allows users to provide their own expression profile in a tab-delimited format. If users would like to replace empirical abundance levels with theoretical models, they may do so by generating their own tab-delimited values. We clarified this point in the “Transcript abundance quantification and simulation” paragraph.
3. Quality of plots used in the manuscript can be improved, preferably to vector quality.
Response: Thanks for pointing this out, as effective and good quality figures are indeed important for effective communication. We recreated all figures in vector quality. In this submission, we also attach all figures in the main text as PDF files.

References
1. Laver, Thomas, et al. "Assessing the performance of the oxford nanopore technologies minion." Biomolecular detection and quantification 3 (2015): 1-8.
2. Loman, Nicholas J., Joshua Quick, and Jared T. Simpson. "A complete bacterial genome assembled de novo using only nanopore sequencing data." Nature methods 12.8 (2015): 733-735.

**Additional Information:**

| Question | Response |
|---|---|
| Are you submitting this manuscript to a special series or article collection? | No |

Powered by Editorial Manager® and ProduXion Manager® from Aries Systems Corporation

| | |
|---|---|
| **Experimental design and statistics**<br><br>Full details of the experimental design and statistical methods used should be given in the Methods section, as detailed in our Minimum Standards Reporting Checklist. Information essential to interpreting the data presented should be made available in the figure legends.<br><br>Have you included all the information requested in your manuscript? | Yes |
| **Resources**<br><br>A description of all resources used, including antibodies, cell lines, animals and software tools, with enough information to allow them to be uniquely identified, should be included in the Methods section. Authors are strongly encouraged to cite Research Resource Identifiers (RRIDs) for antibodies, model organisms and tools, where possible.<br><br>Have you included the information requested as detailed in our Minimum Standards Reporting Checklist? | Yes |
| **Availability of data and materials**<br><br>All datasets and code on which the conclusions of the paper rely must be either included in your submission or deposited in publicly available repositories (where available and ethically appropriate), referencing such data using a unique identifier in the references and in the "Availability of Data and Materials" section of your manuscript.<br><br>Have you have met the above requirement as detailed in our Minimum Standards Reporting Checklist? | Yes |

*Powered by Editorial Manager® and ProduXion Manager® from Aries Systems Corporation*

Manuscript with DOI

Click here to access/download;Manuscript;TNS_GigaScience_manuscript_rev

# Trans-NanoSim characterizes and simulates nanopore RNA-seq data

Saber Hafezqorani[1,2,†], Chen Yang[1,2,†], Theodora Lo[1], Ka Ming Nip[1,2], René L Warren[1], Inanc Birol[1,3,*]

[1] Canada's Michael Smith Genome Sciences Centre, BC Cancer, Vancouver, BC, Canada

[2] Bioinformatics Graduate Program, University of British Columbia, Vancouver, BC, Canada

[3] Department of Medical Genetics, University of British Columbia, Vancouver, BC, Canada

shafezqorani@bcgsc.ca

cheny@bcgsc.ca

tlo@bcgsc.ca

kmnip@bcgsc.ca

rwarren@bcgsc.ca

ibirol@bcgsc.ca

†: Contributed equally

*: Corresponding Author

## Abstract

**Background:** Compared to second-generation sequencing technologies, third-generation single-molecule RNA sequencing has unprecedented advantages; the long reads it generates facilitate isoform-level transcript characterization. In particular, the Oxford Nanopore Technology sequencing platforms have become more popular in recent years due to their relatively high affordability and portability compared to other third-generation sequencing technologies. To aid the development of analytical tools that leverage the

power of this technology, simulated data provides a cost-effective solution with ground truth. However, nanopore sequence simulator targeting transcriptomic data is not available yet.

**Findings:** We introduce Trans-NanoSim, a tool that simulates reads with technical and transcriptome-specific features learnt from nanopore RNA-seq data. We comprehensively benchmarked Trans-NanoSim on direct RNA and cDNA datasets describing human and mouse transcriptomes. Through comparison against other nanopore read simulators, we show the unique advantage and robustness of Trans-NanoSim in capturing the characteristics of nanopore cDNA and direct RNA reads.

**Conclusions:** As a cost-effective alternative to sequencing real transcriptomes, Trans-NanoSim will facilitate the rapid development of analytical tools for nanopore RNA-seq data. Trans-NanoSim and its pre-trained models are freely accessible at https://github.com/bcgsc/NanoSim.

*Keywords:* Nanopore sequencing, Sequence simulation, Transcriptome, RNA-seq

# Findings

RNA-sequencing (RNA-seq) is a cornerstone technology that has helped study and further our understanding of transcriptomes [1]. Third-generation single-molecule sequencing technologies such as those from Oxford Nanopore Technologies (ONT, Oxford, UK) are proving invaluable for isoform-level analyses. For example, ONT reads 1-100 kb in length, permit identification and quantification of most full-length isoforms in the human transcriptome and enable various complex feature analyses [2-5]. In recent years, there has been an increase in the development of novel algorithms to leverage the power of this technology, including *de novo* assembly, alignment and mapping, and structural variant detection [6-12]. In this active field of research, simulated data with a known ground-truth provides a cost-effective means to help develop, refine, and benchmark these tools.

Long-read simulators have been developed for ONT genomic reads [13-14]. DeepSimulator [14] employs a context-dependent deep learning model to simulate the electrical current signals, which are decoded into sequence reads using any off-the-shelf base calling method. Although it may facilitate the development of base calling algorithms, DeepSimulator cannot provide the ground truth at the base level. On the other hand, as a base-level simulator, NanoSim [13] first utilizes statistical models to learn the characteristics of sequencing libraries and then applies those models to simulate ONT genomic reads directly. Although proven to have advanced the development of various bioinformatics analysis tools, NanoSim's initial development was centered on simulating genomic reads [12, 15]. Neither of these tools is specifically designed to capture and reproduce transcriptome-specific features such as transcript expression profiles and intron retention (IR) events. While transcript expression levels inform the biological state of a transcriptome, IR, as one of the main forms of alternative splicing, contributes to the functional complexity of eukaryotic transcriptomes [16]. ONT reads have the potential to capture complex IR events involving multiple introns, thus allowing researchers to investigate IR at isoform-level resolution. In addition, the inadequacy of base callers to detect timespan in the signal data often results in homopolymer expansion

and contraction events, represented by significantly higher deletion rates in homopolymer regions. Despite these homopolymer errors accounting for many, if not majority, of the errors in ONT reads, no ONT read simulator can accurately simulate them. Taking all these into consideration, there is currently an unmet need for an ONT RNA-seq simulator, which can aid the development of transcriptome analysis methods without the expense of sequencing experiments.

Here we present further developments of NanoSim and introduce Trans-NanoSim, which is specifically designed for ONT transcriptome sequencing platform. This versatile tool mimics the technical features of nanopore RNA-Seq data including read error modes, read length distribution and homopolymer artefacts, which might be affected by different library preparation methods and base calling algorithms. Furthermore, Trans-NanoSim can be trained to characterize transcriptome-specific features such as expression patterns and IR events for more accurate simulation. To demonstrate the performance of Trans-NanoSim, we chose three sets of publicly available experimental ONT reads for training and simulation, including human NA12878 direct RNA, cDNA $1D^2$, and mouse cDNA 1D libraries (**Supplementary Note 1**). Through benchmarking the similarity between experimental and simulated reads, we show that Trans-NanoSim consistently outperforms the genomic simulator DeepSimulator, on all three datasets.

Unlike short-reads generated from second-generation sequencing technologies, ONT reads have very long and non-uniform lengths. Thus, read length is a key feature to preserve in simulation. The read length distribution of transcriptomic data is jointly influenced by sequencing techniques, sample preparation protocols (often leading to reads derived from partial transcripts), and transcriptomic variables, such as transcript lengths and expression levels (for the latter, different expression profiles may result in different read length distributions.) Therefore, in order to capture this relationship between expression levels and read lengths, we profiled three datasets and then simulated reads with Trans-NanoSim and DeepSimulator (**Supplementary Note 2**). For the human direct RNA dataset, the length distribution of simulated reads generated by Trans-NanoSim (mean = 807 nt, standard deviation of mean lengths = 0.75 nt determined by ordinary nonparametric bootstrapping 1,000 times using `boot` command in R, **Figure S1**) followed the

empirical read length distribution (mean = 815 nt) closely (**Figure 1A**). Although we configured DeepSimulator to preserve the mean read length of empirical reads (mean = 808 nt), DeepSimulator still generated a bimodal length distribution with a mode of ~150 nt. We suspect that this limitation is due to the predefined read-length distributions of DeepSimulator, while the ONT read length cannot be simply described by a single statistical distribution, as elucidated by previous studies [13]. Further, DeepSimulator, being a genomic read simulator, does not associate the isoform expression levels with read lengths.

Next, we aligned the simulated and empirical reads to the reference genome and evaluated the length of consecutive match/error bases in both sets (**Supplementary Note 2**). While the error rate of the empirical reads from human direct RNA dataset was 10.53%, the simulated reads generated by Trans-NanoSim and DeepSimulator were 10.44% and 11.09%, respectively (**Supplementary Table S1**). Combined with the length distribution of base-calling events, it is evident that Trans-NanoSim mimics error and match events more closely to the experimental data (**Figure 1B**).

For a transcriptome sequence simulator, it is critical to output the correct number of simulated reads for each transcript (i.e., amount that reflects the expected expression level of a given transcript). To evaluate whether a simulated dataset generated by both tools account for transcript isoform usage and expression level, we used the `quantify` module in Trans-NanoSim to compute the transcript expression levels with both empirical and simulated reads (**Supplementary Note 2**). The coefficient of determination ($R^2$) between the estimated transcript abundance of the empirical human direct RNA dataset and the simulated dataset generated by Trans-NanoSim is 0.9444, indicating that the observed raw transcript expression level is well replicated by Trans-NanoSim (**Figure 1C**). In contrast, the $R^2$ value for DeepSimulator simulated reads is 0.0032, which suggests that the transcript abundance in the simulated dataset is independent of its counterpart in the empirical one. Since genomic simulators do not require expression profiles as input, it is expected that this desirable feature is missing.

To the best of our knowledge, Trans-NanoSim is the first transcriptome sequence simulator that provides IR modelling. Considering the human direct RNA dataset as an example, the IR modelling module of Trans-NanoSim identified 2,872 transcripts with at least one retained intron, and nearly half of them (1,285 transcripts) were expressed at over two Transcripts Per Million (TPM). Interestingly, we identified as much as six retained introns in one highly expressed transcript (Ensembl transcript ID: ENST00000425660, TPM = 1,433). The IR modelling module also reports the transitional probability of each intron being retained based on the state of the previous intron, a model that the pipeline uses for read simulations. In the human direct RNA dataset, only 0.41% of reads spanned the first intron of the represented transcript. However, given an intron is retained, the probability of observing the subsequent intron being retained increased to 17.12%.

Another novel feature we introduce to Trans-NanoSim is homopolymer length modelling, which applies to both genome and transcriptome simulations. It is known that the high error rate of ONT reads is partial due to the base calling artefact in homopolymer regions [17] and the base calling errors, majorly deletions, in those regions are substantially higher than in non-homopolymer regions (**Supplementary Table S2**). Trans-NanoSim simulates homopolymer of each base type individually, and in our experiments, the mean homopolymer length is largely consistent between simulated and experimental reads (**Figure 2**). Our analysis revealed a linear correlation between the homopolymer length on the reference compared to the sequencing reads. However, as the homopolymer length increases, less data points were observed, thus widening the confidence interval. As a result, we observed a larger variation between simulated length and experimental lengths for A and T homopolymers longer than 20 nt and C and G homopolymers longer than 15 nt. We note that in the experimental long read datasets used herein, at most only 0.08% and <0.01% of reads containing these homopolymer lengths were observed, respectively and will likely represent rare occurrences in ONT data.

Finally, we evaluated the computational performance of Trans-NanoSim and DeepSimulator through characterizing and simulating 687,192 reads describing the human reference transcriptome

(**Supplementary Note 2**). Although both tools allow users to train a custom model with any dataset, authors of DeepSimulator noted that this step is computationally intensive, and advised their users against trying it [18]. In contrast, in a typical run, it takes Trans-NanoSim less than one hour to train and an additional few minutes to compute the expression profile with four processors. In the simulation stage, Trans-NanoSim ran for 2h11m with peak memory of 526MB, while DeepSimulator ran for 1d8h32m in total (with 5h46m to simulate signals and 1d2h46m for base calling) with peak memory of 17.22 GB (**Supplementary Table S3, S4**). Trans-NanoSim also supports multi-processing, which reduces the runtime significantly, but at the cost of increased memory usage (**Supplementary Figure S2, Table S5**). The runtime of Trans-NanoSim is proportional to the number of reads to be simulated, with a fixed time usage for reading in profiles. The effect of multiprocessing starts to saturate with 12 CPUs when processing less than 60,000 reads, while with more reads, this saturation point is observed with more number of CPUs. Even with only four processors, there is a substantial reduction in runtime (~75% less than the same run on a single CPU), which took 33 minutes to simulate 687,192 human direct RNA reads.

We recapitulated our results by repeating all the analyses presented here on human cDNA $1D^2$ and mouse cDNA sequencing data and obtained similar findings (**Supplementary Figure S3** and **S4**, respectively, and **Table S1**). We noticed that even though the error rates in the raw reads can vary from experiment to experiment, DeepSimulator always generates reads with similar error rates and length distribution, while Trans-NanoSim can adapt to different sequencing libraries and simulates base calling events that are true to the platform.

In this work, we report on results from comprehensive benchmarking experiments to illustrate Trans-NanoSim's performance on three ONT RNA-seq datasets with different sequencing data types: direct RNA, cDNA $1D^2$, and cDNA 1D. Our evaluations demonstrate the robustness of Trans-NanoSim in learning and mimicking the length distribution, sequence error profiles, and homopolymer runs of nanopore RNA-seq reads. Moreover, Trans-NanoSim provides a solution to the characterization of transcriptome-specific features, such as isoform expression and IR events, which cannot be addressed by genomic read simulators.

As a fast and memory-efficient ONT read simulator, Trans-NanoSim is feasible to run on a standard modern-day laptop computer. We anticipate that it will offer an important functionality to the community and it will facilitate the development of various base-level bioinformatics algorithms that leverage the potential of long nanopore reads, including transcriptome assembly, alignment and quantification, structural variant detection, and novel isoform identification.

# Methods

### Trans-NanoSim workflow overview

The workflow of Trans-NanoSim consists of two stages: characterization of experimental reads and simulation from a reference transcriptome (**Figure 3**). In the characterization stage, experimental reads are aligned against the reference transcriptome to infer their source transcript, which is essential for read length analysis and transcript expression quantification. Reads are also aligned against the reference genome to compute statistical models for read error modes. Both genomic and transcriptomic alignments are used to model intron retention events. We also provide pre-trained models along with this work for users to use directly without training. Next, according to these models, reads are simulated given a reference transcriptome and genome. For each read to be simulated, the source reference transcript is selected based on the expression profile. Then, a sequence is extracted from that transcript according to the length distribution model, and it is modified with respect to the IR and error models.

### Length distribution characterization and simulation

Previous versions of NanoSim utilized an empirical cumulative density function to simulate the length distribution of reads. In the current version of the pipeline, NanoSim uses kernel density estimation (KDE), which captures underlying patterns in the read length distributions, and avoids overfitting. We also replace the binning strategy in simulating the alignment ratio on each read with KDE, resulting in a smoother simulated read length distribution. Theoretically, nanopore transcriptome sequencing can yield reads of the

same length as the original mRNA molecule. However, in practice, ONT reads are often shorter than their corresponding mRNA molecules due to experimental or data acquisition artefacts, and thus they may represent partial transcripts. Therefore, it is crucial to consider the length of the reference transcript when simulating the length distribution of simulated ONT reads. In order to achieve this, we utilize a two dimensional KDE model, and measure the length of an ONT read relative to the length of the source transcript. Furthermore, unaligned regions on both ends of each read are also subjected to length distribution analysis. We follow the same KDE model approach as described to model their length distributions separately.

We note that, the percentage of antisense sequences in cDNA and direct RNA sequences may be substantially different. To capture this information, Trans-NanoSim automatically infers the strand ratio by calculating the percentage of reads that are in the same direction as the annotated strand. This strand ratio is then utilized to assign the orientation of reads accordingly during the simulation stage.

**Intron retention characterization and simulation**

Trans-NanoSim is able to detect and model IR events for ONT transcriptome reads. Based on alignments to intronic regions, it uses a Markov chain model to calculate the transitional probabilities between the states of spliced and retained introns, given the state of the previous intron. This feature is not part of the characterization phase by default. To enable this option, transcript annotation file in GTF/GFF format needs to be provided. This functionality can also be invoked in a standalone module (`detect_ir`), enabling users to only detect and model IR events without characterizing or simulating reads. The module outputs comprehensive information on the location of the detected IR events based on input ONT reads.

**Transcript abundance quantification and simulation**

We have incorporated a pipeline [19] to estimate transcript abundance based on reference transcriptome alignments (courtesy of Dr. Jared Simpson, personal communication). The pipeline relies on minimap2 [7]

with `-p0` flag to retain all secondary mappings, and then utilizes an expectation-maximization approach similar to RSEM [20] to assign multi-mapping reads. It is a standalone module (`quantify`) that outputs transcript abundance in TPM values, which can be used in the simulation stage. Users may also provide their own expression profile in tab-delimited format, describing empirical or theoretical distributions, if preferred. During simulation, these transcript abundance values are used to calculate the probability of an isoform being selected and ultimately the number of constituent reads of each isoform.

**Error mode characterization and simulation**

Statistical modeling of error patterns in long nanopore reads was proven to be effective in mimicking the sequencing platform [13]. In Trans-NanoSim, we build on the same mixture models to deal with transcriptome reads as these patterns are shared among different library preparation methods and datasets. According to the alignments, reads are classified into two groups: aligned and unaligned. For each group, we consider specific characterization and modeling approaches. As for the aligned reads, we consider their aligned bases for further error rate analysis. The length of indels and mismatches are drawn from Weibull/Geometric and Poisson/Geometric mixture models, respectively. We also calculate the transitional probability between every two consecutive base call errors using a Markov chain model. We re-implemented the model fitting function of NanoSim in Python (formerly in R), and allowed multi-threading to expedite the fitting process. Unaligned reads may provide crucial information about the nature of ONT sequencing experiments, and thus we chose to model the length distribution of the unaligned reads as well. For this purpose, we extract sequences from reference transcripts based on their length distribution and apply an arbitrarily high error rate (default, 90%). However, since it is impossible to trace their source transcript molecule, unaligned reads are not included in the error rate analysis.

**Homopolymer characterization and simulation**

Previous versions of NanoSim have a k-mer bias parameter (`--k-mer`) in the simulation stage that effectively compresses all homopolymers longer than *n* into *n*-mers. However, it does not simulate

homopolymer expansion events nor is it an accurate representation of the distribution of read homopolymer lengths. In our analysis and the datasets inspected, we observed that the homopolymer length on sequencing reads is consistent with a normal distribution. Further, the mean and associated standard deviation of homopolymer lengths on those same reads is linearly proportional to the reference homopolymer length (**Supplementary Figure S5**). In the simulation stage, Trans-NanoSim first finds homopolymers greater than *n* in the sequence extracted from the reference. Given the reference homopolymer length, the mean and standard deviation, which are used to generate the normal distribution, are calculated from segmented and linear regression models, respectively. The homopolymer length to be simulated is then drawn from the constructed normal distribution, and the extracted sequence is modified accordingly. Depending on the base caller used and sequencing types, the distribution of read homopolymer lengths can vary; thus, we provide pre-trained models to simulate genome and transcriptome reads base called with Albacore, Guppy's default model and Guppy's flip-flop model.

# Availability of supporting source code and requirements

Trans-NanoSim is developed in Python. Source code and pre-trained models for this work are freely accessible at https://github.com/bcgsc/NanoSim (Licence: GPL-3). Trans-NanoSim is also registered in bio.tools (biotools:Trans-NanoSim) and SciCrunch (RRID:SCR_018243) databases.

# Availability of supporting data

Snapshots of our code and other supporting data are openly available in the *GigaScience* repository, GigaDB [21].

# Additional files

Supplementary material

# Abbreviations

| | |
|---|---|
| IR: | Intron Retention |
| KDE: | Kernel Density Estimation |
| ONT: | Oxford Nanopore Technologies |
| RNA-seq: | RNA sequencing |
| TPM: | Transcript Per Million |

# Competing interests

The authors declare that they have no competing interests.

# Funding

This work was supported by Genome Canada and Genome BC [281ANV]; Genome Canada, Genome BC, Genome Quebec, and Genome Alberta [243FOR]; and by the National Human Genome Research Institute of the National Institutes of Health [R01HG007182]. Scholarship funding was provided by the University of British Columbia, and the Natural Sciences and Engineering Research Council of Canada. The content reported is solely the responsibility of the authors, and does not necessarily represent the official views of the funding organizations.

# Author’s contributions

SH and CY contributed equally to this work. IB, SH, and CY conceived and designed the study. SH and CY implemented the algorithm with the help of TL, KMN and RLW. SH drafted and all the other authors reviewed, edited, and approved the final manuscript.

# Acknowledgements

We thank Jared Simpson for his contribution to the transcript expression level quantification module of the pipeline.

# References

1. Wang Z, Gerstein M, Snyder M. RNA-Seq: a revolutionary tool for transcriptomics. Nat Rev Genet. 2009;10:57–63.

2. Oikonomopoulos S, Wang YC, Djambazian H, Badescu D, Ragoussis J. Benchmarking of the Oxford Nanopore MinION sequencing for quantitative and qualitative assessment of cDNA populations. Sci Rep. 2016;6:31602.

3. Garalde DR, Snell EA, Jachimowicz D, Sipos B, Lloyd JH, Bruce M, et al. Highly parallel direct RNA sequencing on an array of nanopores. Nat Methods. 2018;15:201–6.

4. Byrne A, Beaudin AE, Olsen HE, Jain M, Cole C, Palmer T, et al. Nanopore long-read RNAseq reveals widespread transcriptional variation among the surface receptors of individual B cells. Nat Commun. 2017;8:16027.

5. Simpson JT, Workman RE, Zuzarte PC, David M, Dursi LJ, Timp W. Detecting DNA cytosine methylation using nanopore sequencing. Nat Methods. 2017;14:407–10.

6. Loman NJ, Quick J, Simpson JT. A complete bacterial genome assembled de novo using only nanopore sequencing data. Nat Methods. 2015;12:733–5.

7. Li H. Minimap2: pairwise alignment for nucleotide sequences. Bioinformatics. 2018;34:3094–100.

8. Sović I, Šikić M, Wilm A, Fenlon SN, Chen S, Nagarajan N. Fast and sensitive mapping of nanopore sequencing reads with GraphMap. Nat Commun. 2016;7:11307.

9. Jain C, Dilthey A, Koren S, Aluru S, Phillippy AM. A Fast Approximate Algorithm for Mapping Long Reads to Large Reference Databases. J Comput Biol. 2018;25:766–79.

10. Koren S, Walenz BP, Berlin K, Miller JR, Bergman NH, Phillippy AM. Canu: scalable and accurate long-read assembly via adaptive -mer weighting and repeat separation. Genome Res. 2017;27:722–36.

11. Vaser R, Sović I, Nagarajan N, Šikić M. Fast and accurate de novo genome assembly from long uncorrected reads. Genome Res. 2017;27:737–46.

12. Cretu Stancu M, van Roosmalen MJ, Renkens I, Nieboer MM, Middelkamp S, de Ligt J, et al. Mapping and phasing of structural variation in patient genomes using nanopore sequencing. Nat Commun. 2017;8:1326.

13. Yang C, Chu J, Warren RL, Birol I. NanoSim: nanopore sequence read simulator based on statistical characterization. Gigascience. 2017;6:1–6.

14. Li Y, Wang S, Bi C, Qiu Z, Li M, Gao X. DeepSimulator1.5: a more powerful, quicker and lighter simulator for Nanopore sequencing. Bioinformatics. 2020.

15. Marchet C, Morisse P, Lecompte L, Lefebvre A, Lecroq T, Peterlongo P, et al. ELECTOR: evaluator for long reads correction methods. NAR Genom Bioinform. Narnia; 2020

16. Middleton R, Gao D, Thomas A, Singh B, Au A, Wong JJ-L, et al. IRFinder: assessing the impact of intron retention on mammalian gene expression. Genome Biol. 2017;18:51.

17. Weirather JL, de Cesare M, Wang Y, Piazza P, Sebastiano V, Wang X-J, et al. Comprehensive comparison of Pacific Biosciences and Oxford Nanopore Technologies and their applications to transcriptome analysis. F1000Res. 2017;6:100.

18. DeepSimulator Github repository. https://github.com/lykaust15/DeepSimulator. Accessed 29 Jan 2020.

19. The Nanopore RNA Analysis pipeline. https://github.com/jts/nanopore-rna-analysis. Accessed 29 Jan 2020.

20. Li B, Dewey CN. RSEM: accurate transcript quantification from RNA-Seq data with or without a reference genome. BMC Bioinformatics. 2011. Available from: http://dx.doi.org/10.1186/1471-2105-12-323.

21. Hafezqorani S; Yang C; Lo T; Nip KM; Warren RL; Birol I: Supporting data for "Trans-NanoSim characterizes and simulates nanopore RNA-seq data". GigaScience Database. 2020. http://dx.doi.org/10.5524/100750

# Figures

**Figure 1. Benchmarking Trans-NanoSim and DeepSimulator on the human direct RNA dataset**. **A**. Comparison of length distributions of experimental reads and simulated reads generated by Trans-NanoSim and DeepSimulator. **B**. The length of consecutive match/error bases of empirical and simulated reads, as indicated. **C**. Transcript expression levels measured from simulated reads versus the same measured from experimental reads.

**Figure 2. Homopolymer simulation performance on the human direct RNA dataset.** The x-axis shows the reference homopolymer length (nt) and y-axis is the mean homopolymer length (nt) on corresponding reads. The distributions for A and T homopolymers are trimmed at 40 nt.

**Figure 3. Schematic overview of the Trans-NanoSim pipeline.** The first stage (Characterization) of the pipeline aligns input ONT transcriptome reads against the reference transcriptome and genome to statistically model the read length distribution and error modes. It also optionally detects intron retention events and quantifies transcript expression. These profiles alongside homopolymer model are then used in the second stage (Simulation) to generate simulated reads, also reporting their associated error profiles.

Figure 1

Click here to access/download;Figure;Fig1-14Apr20.pdf

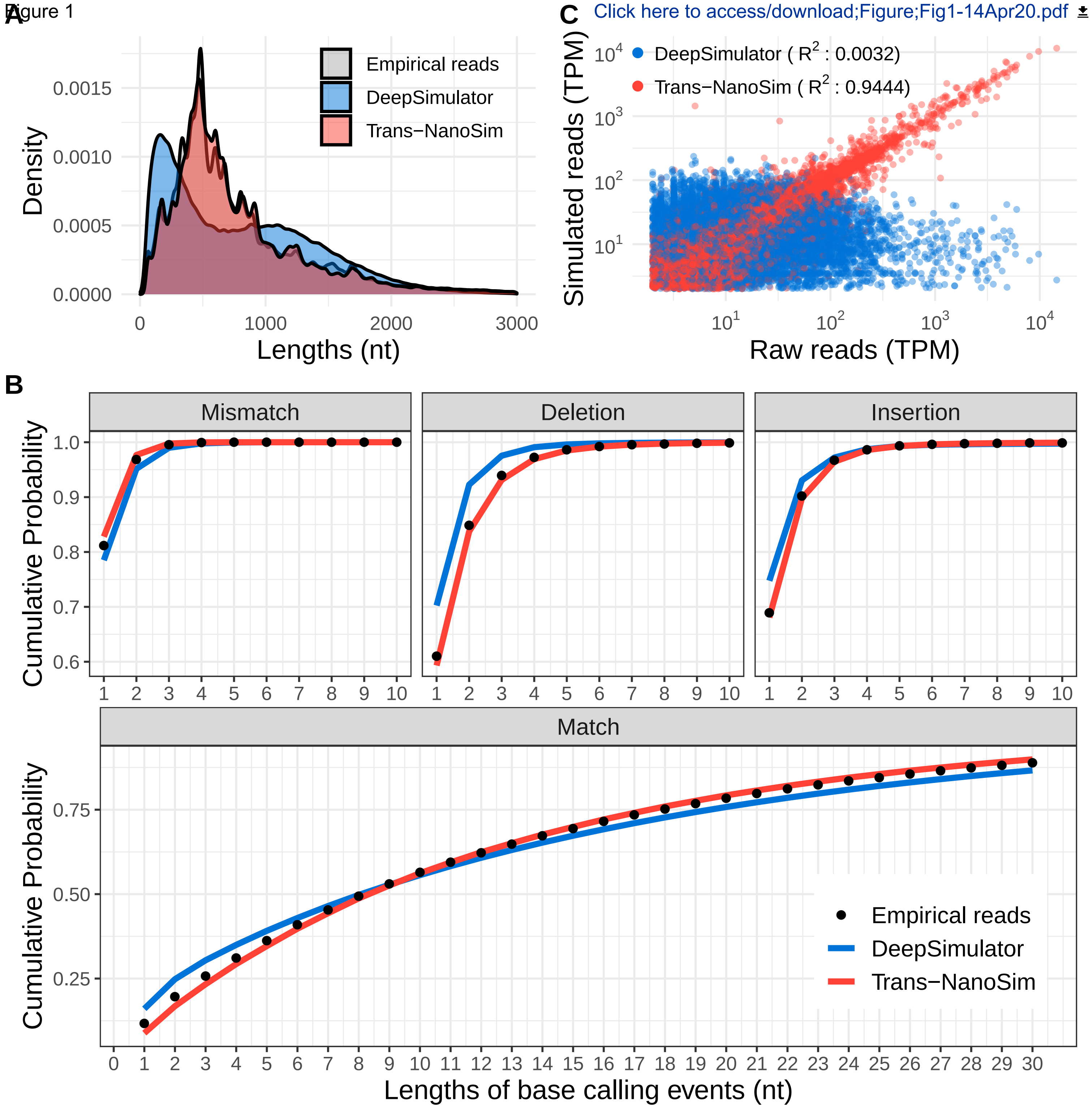

Figure 2

Click here to access/download;Figure;Fig2-14Apr20.pdf

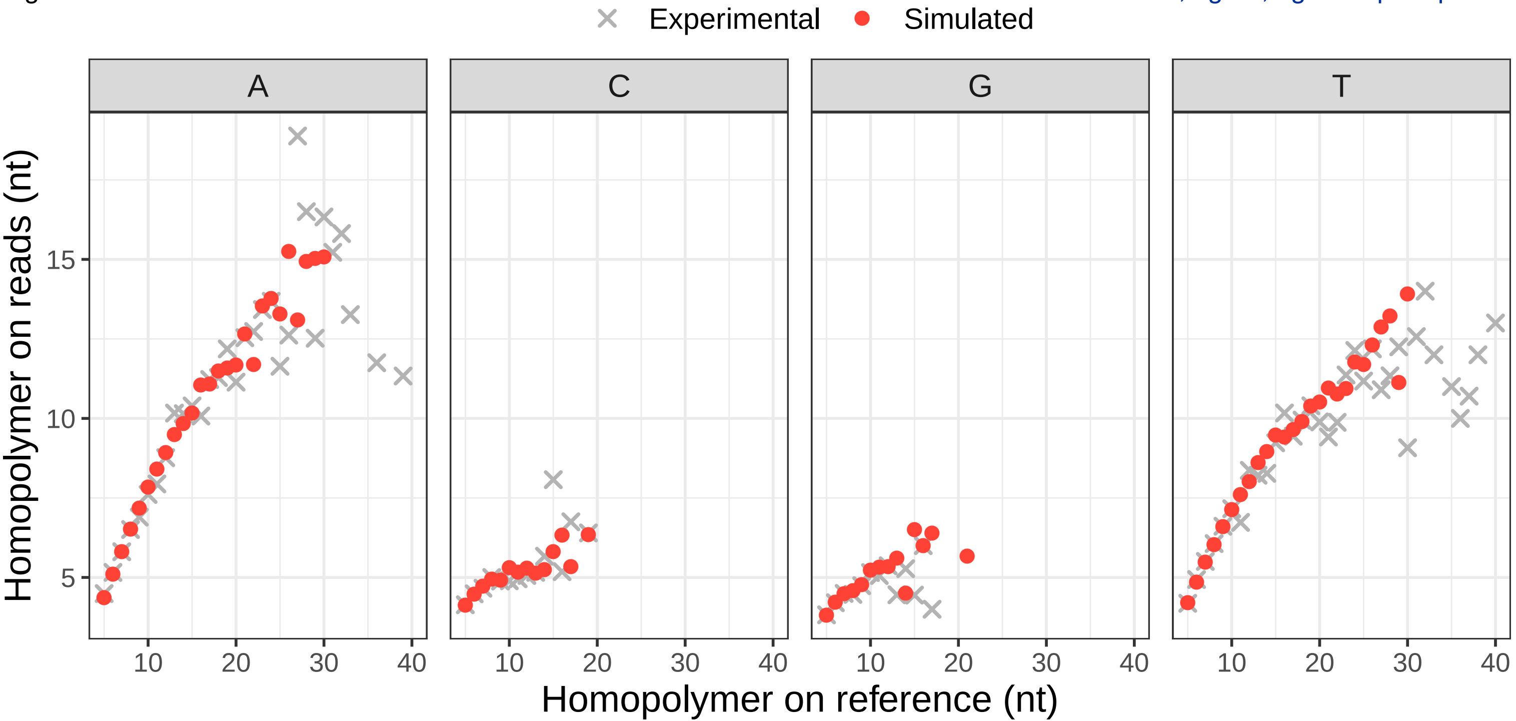

Figure 3

Click here to access/download;Figure;Fig3-14Apr20.pdf

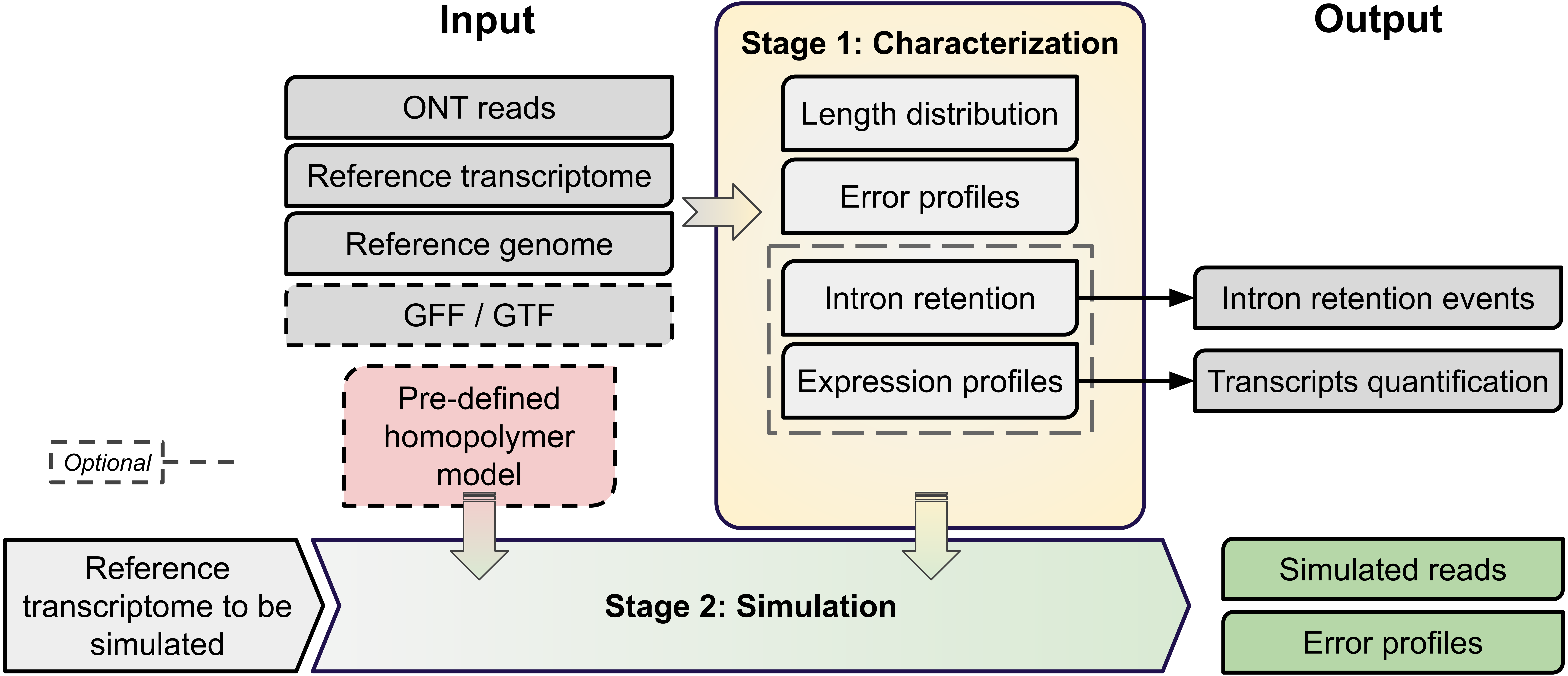

Click here to access/download
**Supplementary Material**
TNS_GigaScience_supplementary_revision_14Apr20.pd
f

 Click here to access/download;Personal Cover;TNS_GigaScience_reviewer_comments_14Apr20.docx

Dear Dr. Nogoy,

Thank you for your consideration of our manuscript. We appreciate the thorough and constructive comments from you and our reviewers. In our revised submission, we have addressed the concerns raised, as outlined below, and edited the manuscript accordingly. We also registered Trans-NanoSim in bio.tools and SciCrunch databases and included the identifiers in the manuscript as requested.

Sincerely,

Saber Hafezqorani, Chen Yang, and Inanc Birol
Canada's Michael Smith Genome Sciences Centre
British Columbia Cancer Agency

REVIEWER 1

1. **"The alignment experimental reads against the reference transcriptome and genome" I would like to clarify whether this order is 1/ transcriptome then 2/ genome here?**

**Response**: We align each read set to both reference transcriptome and to reference genome. The genome alignments are used to detect retained introns, so we can compute the error rates more accurately. The transcriptome alignments are used to assign the source transcript for each read, which is essential for read length distribution analysis and transcript expression level quantification. Both genome and transcriptome alignments are required to model intron retention events. In other words, the alignment order has no effect on the characterization phase. We have revised the text in the 1st paragraph in Methods to clarify this issue.

2. **A "staircase effect" can be observed because of truncated reads in ONT experiment. I could not see a mention to truncated reads nor to this effect in the article, however I think this can be an important one. Can this be modeled within the length distribution?**

**Response**: As noted, nanopore reads are often shorter than their corresponding mRNA molecules due to experimental or data acquisition artefacts, and thus they may represent partial transcripts. In our revised manuscript, we further clarified and explained how we consider this in our analysis.

Trans-NanoSim models the length distribution of nanopore RNA-seq reads based on the primary alignment of these reads to the reference transcriptome. During simulation, a transcript is selected from the expression profiles and read lengths are then selected from the read length distribution models to generate synthetic reads from different parts of that given transcript. We modified the following sections of our manuscript to clarify this issue:

- 4$^{th}$ paragraph of Findings section: We mention several reasons that cause nanopore reads to be often shorter than their corresponding mRNA molecules.
- "Length distribution characterization and simulation" - Methods section: We clarify that reads with varying lengths may be derived from different parts of a given transcript.

3. **The unaligned bases are not taken into account to estimate the error rate (though the unaligned reads are used to determine the length distribution). This is a (reasonable) choice, which limitations should be clearly discussed.**

**Response**: To calculate the error rate or accuracy for ONT reads in Trans-NanoSim, the aligned length of query sequence is used as the denominator. We note that, for this definition we are following the convention set in other studies [1, 2]. Our justification is, since the source transcript molecule is unknown for unaligned reads, it is not possible to include unaligned reads for error rate estimation. The limitation of this formula is that the error rate could be slightly inflated. We added a sentence at the 6$^{th}$ paragraph of "Method" section to clarify this.

4. **"It is observed that the homopolymer lengths on reads follow normal distribution [...]". Do you have other sources you could cite about this?**

**Response**: This statement is based on our own analysis, and we edited the "Homopolymer characterization and simulation" paragraph to clarify it. We also describe this analysis at 8$^{th}$ paragraph of "Findings" section (homopolymer modeling). The following sentences are from that paragraph:

"Trans-NanoSim simulates homopolymer of each base type individually, and in our experiments, the mean homopolymer length is largely consistent between simulated and experimental reads (Figure 2). Our analysis revealed a linear correlation between the homopolymer length on the reference compared to the sequencing reads."

5. **I think sometimes the text contains too many repetitions. For instance, " In this work, we introduce the first ONT transcriptome sequence simulator [...]" could be removed as this has already been clearly stated before.**

**Response**: Thanks for the constructive comment to improve the readability of the text. In this revision, we thoroughly reviewed the manuscript to increase its readability and removed the repetitive phrases.

REVIEWER 2

Major comment:

1. **My main suggestion is to add a comparison against IsoSeqSim (https://github.com/yunhaowang/IsoSeqSim). Although it was released quite some time ago and may not have such functionality as introducing intron retention, it still has main simulation features such as truncating transcripts and introducing errors according to given profiles.**

**Response**: We thank the reviewer for bringing this tool to our attention. After careful thought and in communication with our editor we decided not to include benchmarks against IsoSeqSim. While we do not have any reason to doubt the validity of the approach implemented in IsoSeqSim, we note that it neither has a peer-reviewed publication nor a preprint describing the work. Further, we cannot determine its usage in other studies, hence its impact in the field. At the time of this writing (March 27, 2020), a Google search for “IsoSeqSim” returned only four hits, all of which were pages created by the maintainer of IsoSeqSim. According to the method’s GitHub page (https://github.com/yunhaowang/IsoSeqSim), the repository has not been updated for at least two years. We also do not see any commits, forks, pull-requests, stars, or issues (opened or closed) from other users. Thus, we conclude that IsoSeqSim is not a mature enough tool to count as being part of the state-of-the-art.

Minor comments:

1. **Would be useful to provide some trained models for 1-2 typical ONT experiments in the package to allow a user to make a quick start.**

**Response**: We totally agree that providing pre-trained models would be beneficial for users to make a quick start. In this regard, we provide several models for users to use directly without training. We mention this throughout the manuscript:

- *Conclusion* section in “Abstract”
- *Trans-NanoSim workflow overview* section in “Methods”
- *Availability of supporting source code and requirements* section

2. **For the same reason, it could be helpful to add the possibility to simulate reads without transcript abundance file. Instead of real abundances, one can use some approximation (e.g. negative binomial distribution).**

**Response**: We thank the reviewer for this suggestion. We agree that the transcript expression levels change between different experiments, and users may want to test different scenarios. We note that, Trans-NanoSim is quite flexible in this regard; it allows users to provide their own expression profile in a tab-delimited format. If users would like to replace empirical abundance levels with theoretical models, they may do so by generating their own tab-delimited values. We clarified this point in the “Transcript abundance quantification and simulation” paragraph.

3. **Quality of plots used in the manuscript can be improved, preferably to vector quality.**

**Response**: Thanks for pointing this out, as effective and good quality figures are indeed important for effective communication. We recreated all figures in vector quality. In this submission, we also attach all figures in the main text as PDF files.

**References**

1. Laver, Thomas, et al. "Assessing the performance of the oxford nanopore technologies minion." *Biomolecular detection and quantification* 3 (2015): 1-8.

2. Loman, Nicholas J., Joshua Quick, and Jared T. Simpson. "A complete bacterial genome assembled de novo using only nanopore sequencing data." *Nature methods* 12.8 (2015): 733-735.
