# Supplementary material for: Trans-NanoSim characterizes and simulates nanopore RNA-sequencing data
Source: Gigascience. 2020 Jun 10;9(6):giaa061. doi: 10.1093/gigascience/giaa061 (PMC7285873; doi:10.1093/gigascience/giaa061)
Supplement: giaa061_Supplemental_File [file giaa061_supplemental_file.pdf]

Supplementary information for "Trans-NanoSim characterizes and simulates nanopore RNA-seq data"

Saber Hafezqorani, Chen Yang, Theodora Lo, Ka Ming Nip, René L Warren, Inanc Birol

Supplementary Note 1: Datasets

We used publicly available direct RNA and cDNA Oxford Nanopore Technology (ONT) sequencing reads describing human and mouse transcriptomes provided by the Nanopore WGS Consortium RNA project [1] and Byrne *et al.* [2], respectively. From the Nanopore WGS Consortium – RNA project there are 30 runs (flowcells) of direct RNA reads sequenced on the Oxford Nanopore MinION device from 6 different centres. In addition, there are 12 cDNA read datasets sequenced using 1D ligation kit (SQK-LSK108) as well as $1D^2$ ligation kit (SQK-LSK308) using the R9.4 chemistry. We also downloaded and used cDNA sequences from mouse transcriptome provided in the Byrne et al manuscript. We trained/benchmarked our tool using read sets provided in these two data sources. In particular, we analyzed the following three read sets from those studies. For each dataset, we used high quality reads filtered by NanoFilt [3] for training and simulation. For the benefit of the community and the sake of reproducibility of our results, we provide the pre-trained models on the NanoSim Github repository (https://github.com/bcgsc/NanoSim).

1. Human direct RNA dataset

- Centre: Birmingham (Bham) – Run 1
- Sample Type: RNA
- Kit: SQK-RNA001
- Pore Chemistry: R9.4
- Base caller: Guppy CPU (v3.1.5)
- Number of reads: 1,076,330 (687,192 after filtering with NanoFilt with minimum quality score of 7 and minimum length of 50 nt)
- Reference manuscript: https://www.nature.com/articles/s41592-019-0617-2
- Reference transcriptome (annotation): Homo_sapiens.GRCh38.cdna.all.fa (Ensembl 90)

2. Human cDNA dataset

- Centre: Birmingham (Bham) – Run 1
- Sample Type: $1D^2$ cDNA
- Kit: SQK-LSK308
- Pore Chemistry: R9.4
- Base caller: Albacore (v2.1)
- Number of reads: 2,530,461 (728,689 after filtering with NanoFilt with minimum quality score of 7 and minimum length of 50 nt)
- Reference manuscript: https://www.nature.com/articles/s41592-019-0617-2

- Reference transcriptome (annotation): Homo_sapiens.GRCh38.cdna.all.fa (Ensembl 90)

3. Mouse cDNA dataset

- Experiment: SRR5286960
- Sample Type: 1D cDNA
- Pore Chemistry: R9.4
- Base caller: Cloud-based Metrichor
- Number of reads: 104,990 (104,979 after filtering with NanoFilt with minimum quality score of 7 and minimum length of 50 nt)
- Reference manuscript: https://www.nature.com/articles/ncomms16027
- Reference transcriptome (annotation): Mus_musculus.GRCm38.cdna.all.fa (Ensembl 91)

Supplementary Note 2: Simulating reads from human and mouse reference transcriptomes and analyses

Benchmark analyses of Trans-NanoSim and DeepSimulator were performed on machines running on CentOS 6.7 with 48 Intel Xeon E5-2650 CPUs and 380 GB of memory.

Trans-NanoSim: For each experiment, we used the empirical reads mentioned in **Supplementary Note 1** to train Trans-NanoSim. In addition, we used the mouse and human reference transcriptomes and genomes in the characterization phase. By default, Trans-NanoSim uses minimap2 [4] (v2.17-r941 in this work) to align reads to both reference transcriptome and genome for each species. Later, based on these alignments, Trans-NanoSim calculates the length distribution of reads as well as their error profiles. We used the `quantify` module of the pipeline to quantify expression levels of the Ensembl annotated transcripts. Next, in the simulation phase, these profiles are used to generate the same number of simulated reads as the number of raw reads. All the benchmark results presented in this manuscript are done using Trans-NanoSim v2.5.1. Detailed information about parameter settings and input files used in each step is as follows:

1. Characterization (`read_analysis.py transcriptome`):
    - `-i`: Input ONT reads in FASTQ/FASTA
    - `-rg`: Reference genome in FASTA
    - `-rt`: Reference transcriptome in FASTA
    - `-annot`: Annotation file in Ensembl GTF/GFF
2. Quantification (`read_analysis.py quantify`):
    - `-i`: Input ONT reads in FASTQ/FASTA
    - `-rt`: Reference transcriptome in FASTA
3. Simulation (`simulator.py transcriptome`):
    - `-rt`: Reference transcriptome in FASTA
    - `-rg`: Reference genome in FASTA
    - `-e`: Expression profiles in TSV format (from quantification)
    - `-c`: Location and prefix of read profiles (from characterization)
    - `-n`: Number of reads to simulate

Additionally, we used `-k 6 -b guppy -r dRNA` for homopolymer simulation (**Figure 2**). We used `--no_model_ir` to skip intron retention simulation in all experiments except for runtime/memory comparisons (**Table S3-5**).

For the full set of options available in each step and example runs, users may refer to the comprehensive readme file in GitHub repository: https://github.com/bcgsc/NanoSim

Parameter settings for minimap2 within Trans-NanoSim:

- Alignment to reference transcriptome:
    - `minimap2 --cs -ax map-ont`
- Alignment to reference genome:
    - `minimap2 --cs -ax splice`

DeepSimulator: We downloaded and used the latest released version of DeepSimulator (v1.5) from the GitHub repository (https://github.com/lykaust15/DeepSimulator). The reference transcriptome was used as input. DeepSimulator allows users to specify the average length of the reads to be simulated. For a fair comparison, the average length of empirical ONT transcriptome reads (**Supplementary Note 1**) was provided through the `-l` option to preserve the mean length in the simulated reads. We used Guppy CPU (v3.1.5) to base call signals for the human direct RNA simulated reads and Albacore (v2.3.1) for the human cDNA simulated reads because the empirical reads were base called by the respective base callers. The mouse cDNA empirical reads were base called by Metrichor. Since Metrichor is not publicly available anymore, we had to use DeepSimulator's default choice, Guppy (v3.1.5) for this analysis. Other parameters such as the length distribution pattern were kept as default. We simulated the same number of simulated reads as the number of raw reads. Detailed information about parameter settings and input files is as follows:

- `-i`: Reference transcriptome in FASTA
- `-o`: Output directory
- `-B`: Base caller to be used
- `-l`: Mean length of reads to be simulated
- `-n`: Number of simulated reads

Base call analyses for simulated and raw reads:

Parameter settings for aligning raw and simulated reads to reference genome:

```
minimap2 --cs -ax splice
```

For each read, the `cs` tag from the primary alignment was parsed to record the stretches of correct bases and the mismatch, insertion and deletion errors. Accumulatively, these values from all reads are used to calculate to overall matching rate and error rates for each dataset.

Supplementary Tables

For the following tables, we abbreviated simulated reads from Trans-NanoSim as "TNS", simulated reads from DeepSimulator as "DS", and raw experimental reads as "Raw".

Table S1. Error rates in empirical and simulated reads. We aligned the simulated reads generated by Trans-NanoSim and DeepSimulator back to the reference genome and evaluated the rate of error events in simulated reads and raw experimental read counterparts.

% of the all bases having	Human cDNA			Human direct RNA			Mouse cDNA		
	Raw	TNS	DS	Raw	TNS	DS	Raw	TNS	DS
**Mismatch**	4.77	4.53	2.48	2.61	2.63	3.51	1.62	1.60	3.45
**Insertion**	7.22	6.68	6.00	2.30	2.22	4.37	1.22	1.13	6.13
**Deletion**	5.07	4.53	2.28	5.61	5.58	3.20	5.35	5.55	3.03
**Total**	17.07	15.75	10.77	10.53	10.44	11.09	8.21	8.29	12.61

Table S2. Error rates in homopolymer regions and non-homopolymer regions for human direct RNA dataset.

Error type	Insertion	Mismatch	Deletion	Total
**Homopolymer**	0.02233	0.02579	0.16627	0.21439
**Non-homopolymer**	0.02287	0.02495	0.05103	0.09885

Table S3. Runtime usage in simulating Human direct RNA dataset for Trans-NanoSim and DeepSimulator. The reported time is wallclock time in hh:mm:ss format. For DeepSimulator, the runtime comes in two parts, signal generation + base calling. The best runtime for each simulated set is bolded.

Number of reads	10,000	50,000	100,000	687,192
**TNS**	00:02:22	00:08:49	00:17:09	02:11:30
**TNS + IR**	00:04:44	00:11:46	00:21:09	02:21:44
**TNS 4 processors**	**00:01:16**	**00:03:05**	**00:05:17**	**00:33:35**
**DS + Guppy**	00:06:06 + 00:24:28	00:25:35 + 02:06:39	00:53:23 + 03:57:04	05:46:34 + 26:46:39

Table S4. Memory usage (maximum resident set size in GB) in simulating Human direct RNA dataset for Trans-NanoSim and DeepSimulator. The reported memory is maximum resident set size. The lowest memory usage for each simulated set is bolded.

Number of reads	10,000	50,000	100,000	687,192
**TNS**	**0.50**	**0.49**	**0.53**	**0.52**
**TNS + IR**	0.97	0.98	0.95	1.01
**TNS 4 processors**	2.46	2.45	2.68	2.51
**DS + Guppy**	17.22	17.22	17.22	17.22

Table S5. Trans-NanoSim multiprocessing memory usage (maximum resident set size in GB) with IR modelling.

Reads \ Processors	1	4	8	12
**20,000**	0.98	4.85	8.47	12.32
**40,000**	1.00	4.53	8.60	11.82
**60,000**	0.99	4.89	8.27	12.14
**80,000**	0.96	4.79	8.26	11.91
**100,000**	0.98	4.92	8.80	11.98

Supplemental Figures

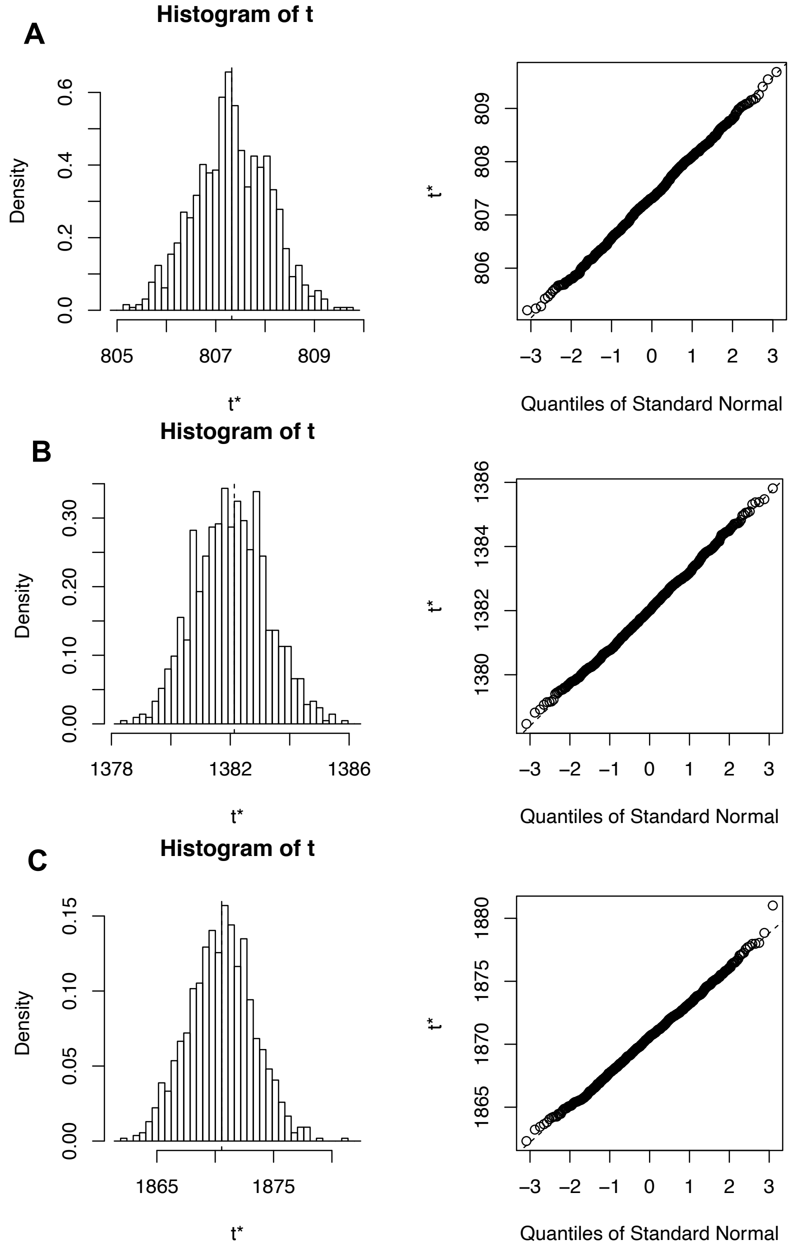

Figure S1. Bootstrapping results for length distribution analyses. The x-axis in the left plots (t*) is the average length of reads in each bootstrapping sample set. **A**. Human direct RNA dataset. The average length of the original read set is 807 nt (Standard deviation (Std) = 0.75). The 95% confidence interval is between 805 and 808 nt. **B**. Human cDNA dataset. The average length of the original read set is 1,382 nt (Std = 1.20). The 95% confidence interval is between 1,380 and 1,385. **C**. Mouse cDNA dataset. The average length of the original read set is 1,870 nt (Std = 2.76). The 95% confidence interval is between 1,865 and 1,876.

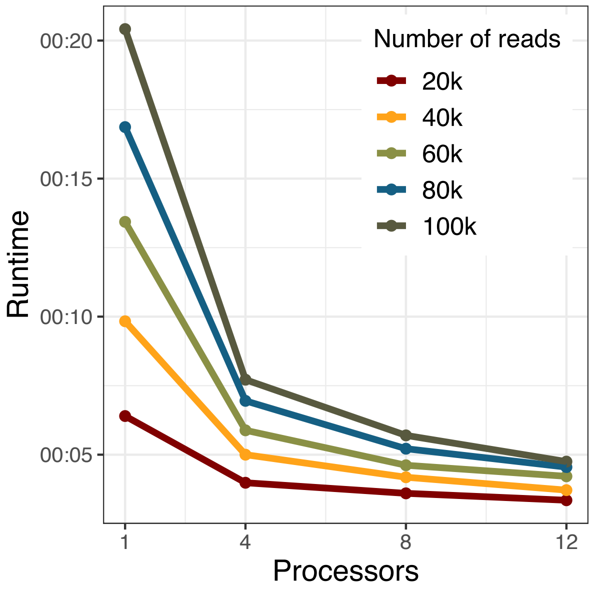

Figure S2. Runtimes for multiprocessing. Wallclock time (hh:mm) for Trans-NanoSim simulation stage with 1, 4, 8, and 12 processors.

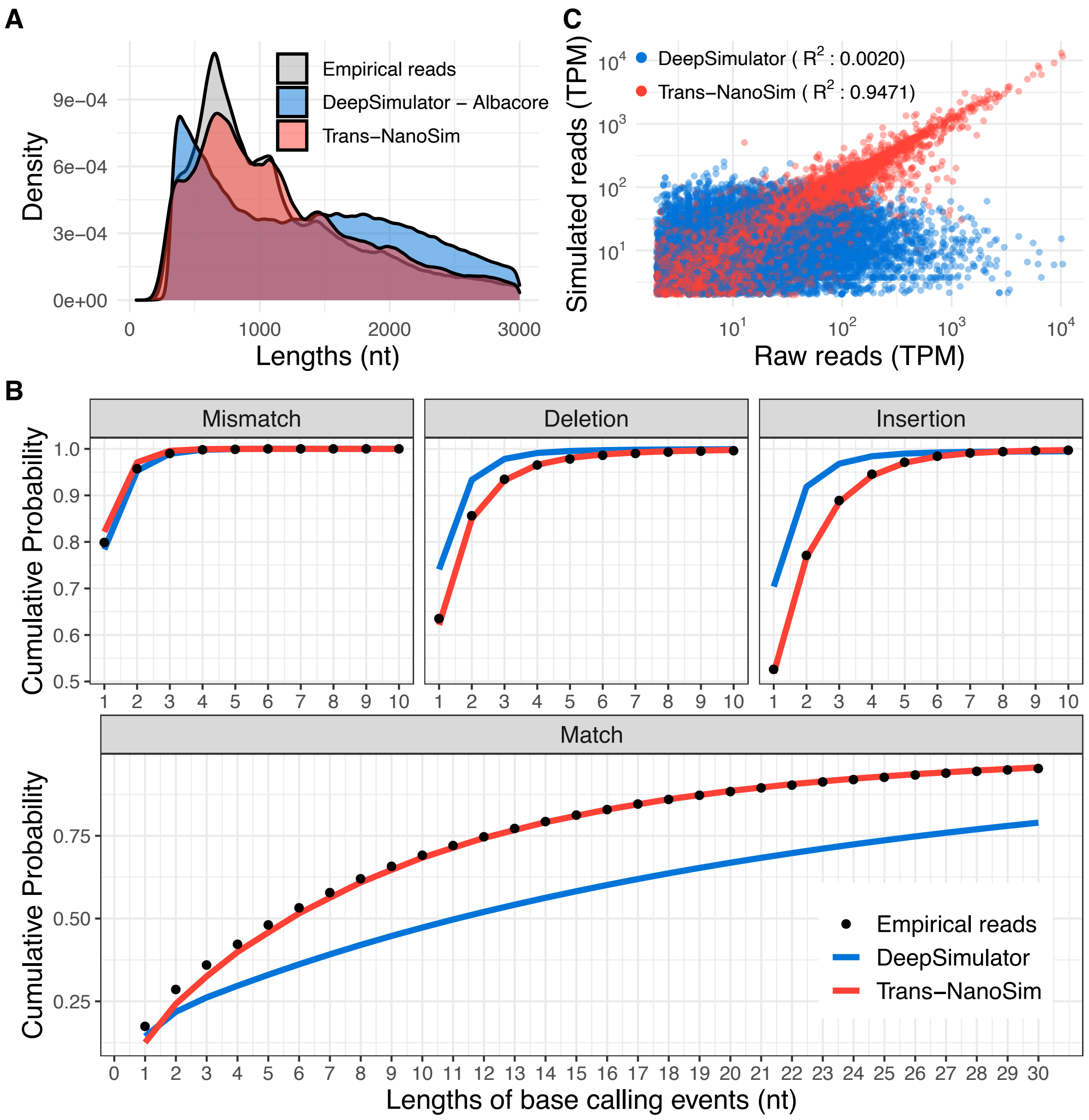

Figure S3. Benchmarking Trans-NanoSim and DeepSimulator on the human cDNA 1D^2 dataset. A. Comparison of length distributions of experimental reads and simulated reads generated by Trans-NanoSim and DeepSimulator. **B**. The length of consecutive match/error bases of empirical and simulated reads, as indicated. **C**. Transcript expression levels measured from simulated reads versus the same measured from experimental reads.

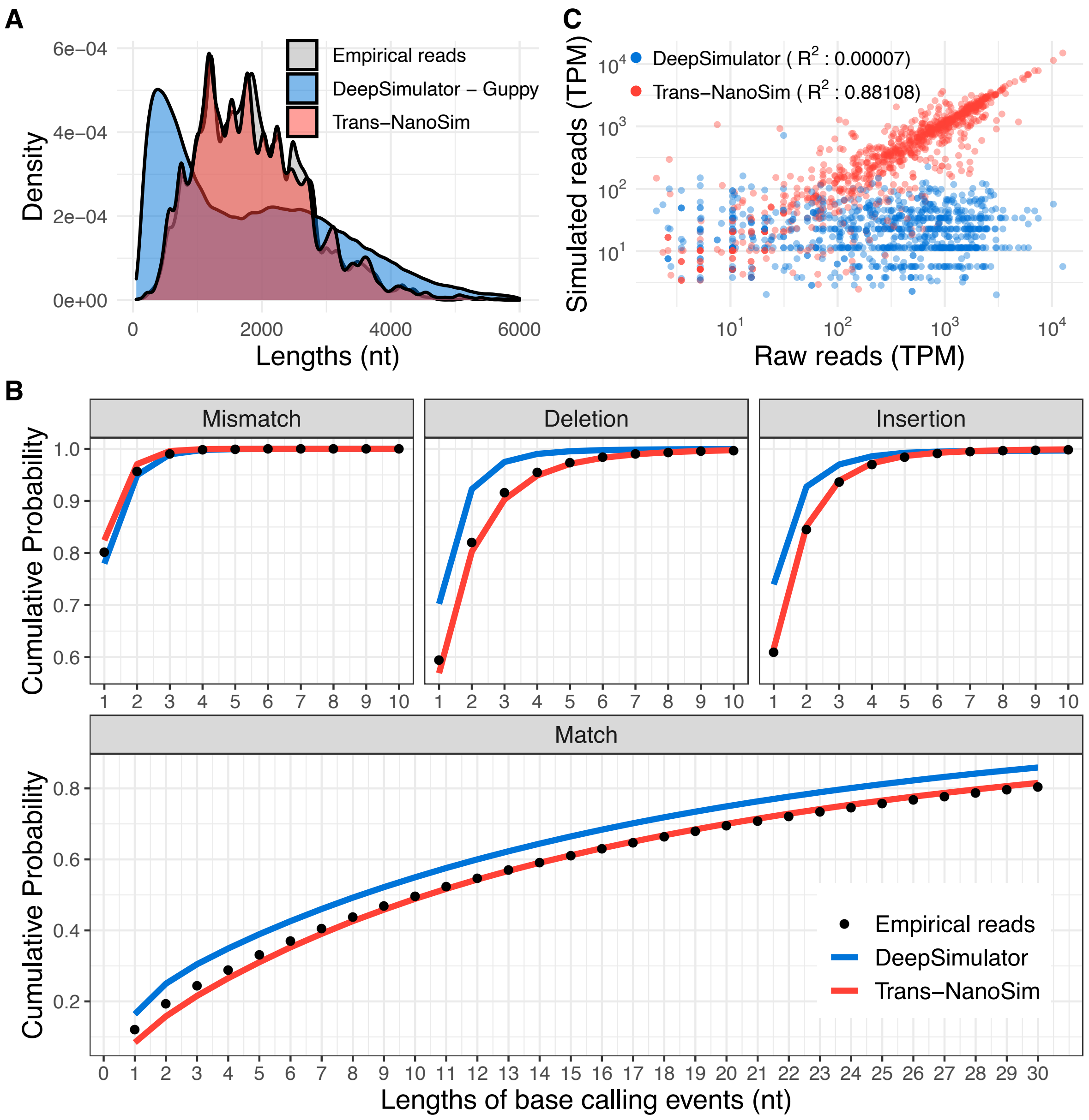

Figure S4. Benchmarking Trans-NanoSim and DeepSimulator on the mouse cDNA dataset. A. Comparison of length distributions of experimental reads and simulated reads generated by Trans-NanoSim and DeepSimulator. **B**. The length of consecutive match/error bases of empirical and simulated reads, as indicated. **C**. Transcript expression levels measured from simulated reads versus the same measured from experimental reads.

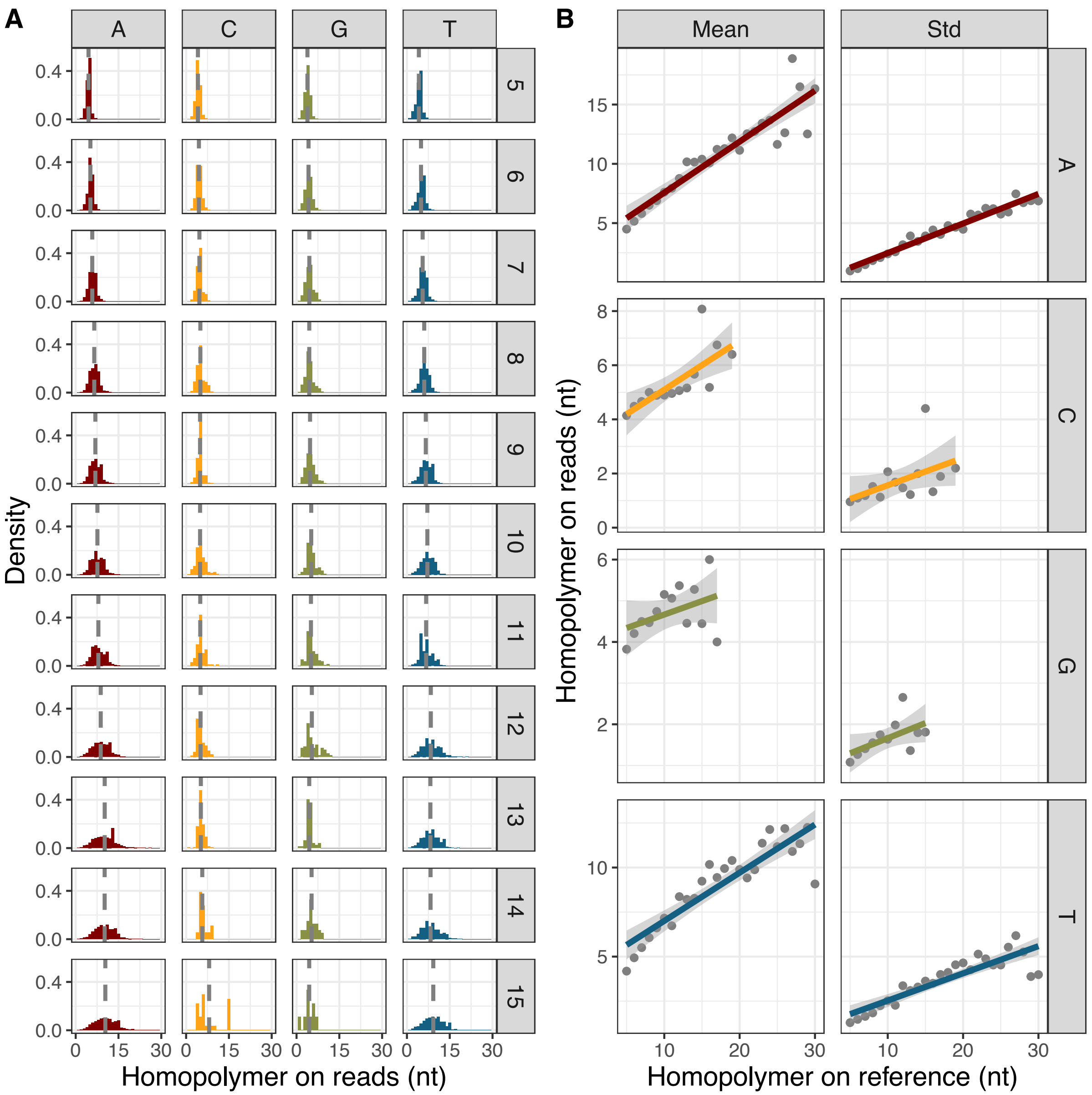

Figure S5. Homopolymer characterization of human NA12878 direct RNA dataset. A. Each facet represents one homopolymer (length <= 15 nt) on the reference transcriptome, the x-axis of which designates the corresponding homopolymer length (<= 30 nt) on the raw reads. **B**. The y-axis represents the mean homopolymer length on raw reads. The fitted line is calculated based on linear regression and the grey area represents the 95% confidence interval.

Supplementary References

1. Workman RE, Tang AD, Tang PS, Jain M, Tyson JR, Razaghi R, et al. Nanopore native RNA sequencing of a human poly(A) transcriptome. Nat Methods. 2019;16:1297–305.

2. Byrne A, Beaudin AE, Olsen HE, Jain M, Cole C, Palmer T, et al. Nanopore long-read RNAseq reveals widespread transcriptional variation among the surface receptors of individual B cells. Nat Commun. 2017;8:16027.

3. De Coster W, D'Hert S, Schultz DT, Cruts M, Van Broeckhoven C. NanoPack: visualizing and processing long-read sequencing data. Bioinformatics. 2018;34:2666–9.

4. Li H. Minimap2: pairwise alignment for nucleotide sequences. Bioinformatics. 2018;34:3094–100.
