# Supplementary material for: Trans-NanoSim characterizes and simulates nanopore RNA-sequencing data
Source: Gigascience. 2020 Jun 10;9(6):giaa061. doi: 10.1093/gigascience/giaa061 (PMC7285873; doi:10.1093/gigascience/giaa061)
Supplement: giaa061_Response_to_Reviewer_Comments_Original_Submission [file giaa061_response_to_reviewer_comments_original_submission.pdf]

Author's Response To Reviewer Comments

Close

[A formatted version of our Response to Reviewers is attached as our cover letter]

Dear Dr. Nogoy,
Thank you for your consideration of our manuscript. We appreciate the thorough and constructive comments from you and our reviewers. In our revised submission, we have addressed the concerns raised, as outlined below, and edited the manuscript accordingly. We also registered Trans-NanoSim in bio.tools and SciCrunch databases and included the identifiers in the manuscript as requested.
Sincerely,
Saber Hafezqorani, Chen Yang, and Inanc Birol
Canada's Michael Smith Genome Sciences Centre
British Columbia Cancer Agency

REVIEWER 1
1. "The alignment experimental reads against the reference transcriptome and genome" I would like to clarify whether this order is 1/ transcriptome then 2/ genome here?
Response: We align each read set to both reference transcriptome and to reference genome. The genome alignments are used to detect retained introns, so we can compute the error rates more accurately. The transcriptome alignments are used to assign the source transcript for each read, which is essential for read length distribution analysis and transcript expression level quantification. Both genome and transcriptome alignments are required to model intron retention events. In other words, the alignment order has no effect on the characterization phase. We have revised the text in the 1st paragraph in Methods to clarify this issue.
2. A "staircase effect" can be observed because of truncated reads in ONT experiment. I could not see a mention to truncated reads nor to this effect in the article, however I think this can be an important one. Can this be modeled within the length distribution?
Response: As noted, nanopore reads are often shorter than their corresponding mRNA molecules due to experimental or data acquisition artefacts, and thus they may represent partial transcripts. In our revised manuscript, we further clarified and explained how we consider this in our analysis.
Trans-NanoSim models the length distribution of nanopore RNA-seq reads based on the primary alignment of these reads to the reference transcriptome. During simulation, a transcript is selected from the expression profiles and read lengths are then selected from the read length distribution models to generate synthetic reads from different parts of that given transcript. We modified the following sections of our manuscript to clarify this issue:
• 4th paragraph of Findings section: We mention several reasons that cause nanopore reads to be often shorter than their corresponding mRNA molecules.
• "Length distribution characterization and simulation" - Methods section: We clarify that reads with varying lengths may be derived from different parts of a given transcript.
3. The unaligned bases are not taken into account to estimate the error rate (though the unaligned reads are used to determine the length distribution). This is a (reasonable) choice, which limitations should be clearly discussed.
Response: To calculate the error rate or accuracy for ONT reads in Trans-NanoSim, the aligned length of query sequence is used as the denominator. We note that, for this definition we are following the convention set in other studies [1, 2]. Our justification is, since the source transcript molecule is unknown for unaligned reads, it is not possible to include unaligned reads for error rate estimation. The limitation of this formula is that the error rate could be slightly inflated. We added a sentence at the 6th paragraph of "Method" section to clarify this.
4. "It is observed that the homopolymer lengths on reads follow normal distribution [...]". Do you have other sources you could cite about this?
Response: This statement is based on our own analysis, and we edited the "Homopolymer characterization and simulation" paragraph to clarify it. We also describe this analysis at 8th paragraph of "Findings" section (homopolymer modeling). The following sentences are from that paragraph: "Trans-NanoSim simulates homopolymer of each base type individually, and in our experiments, the mean homopolymer length is largely consistent between simulated and experimental reads (Figure 2).

Our analysis revealed a linear correlation between the homopolymer length on the reference compared to the sequencing reads.”
5. I think sometimes the text contains too many repetitions. For instance, " In this work, we introduce the first ONT transcriptome sequence simulator [...]" could be removed as this has already been clearly stated before.
Response: Thanks for the constructive comment to improve the readability of the text. In this revision, we thoroughly reviewed the manuscript to increase its readability and removed the repetitive phrases.
REVIEWER 2
Major comment:
1. My main suggestion is to add a comparison against IsoSeqSim (https://github.com/yunhaowang/IsoSeqSim). Although it was released quite some time ago and may not have such functionality as introducing intron retention, it still has main simulation features such as truncating transcripts and introducing errors according to given profiles.
Response: We thank the reviewer for bringing this tool to our attention. After careful thought and in communication with our editor we decided not to include benchmarks against IsoSeqSim. While we do not have any reason to doubt the validity of the approach implemented in IsoSeqSim, we note that it neither has a peer-reviewed publication nor a preprint describing the work. Further, we cannot determine its usage in other studies, hence its impact in the field. At the time of this writing (March 27, 2020), a Google search for “IsoSeqSim” returned only four hits, all of which were pages created by the maintainer of IsoSeqSim. According to the method’s GitHub page (https://github.com/yunhaowang/IsoSeqSim), the repository has not been updated for at least two years. We also do not see any commits, forks, pull-requests, stars, or issues (opened or closed) from other users. Thus, we conclude that IsoSeqSim is not a mature enough tool to count as being part of the state-of-the-art.
Minor comments:
1. Would be useful to provide some trained models for 1-2 typical ONT experiments in the package to allow a user to make a quick start.
Response: We totally agree that providing pre-trained models would be beneficial for users to make a quick start. In this regard, we provide several models for users to use directly without training. We mention this throughout the manuscript:
• Conclusion section in “Abstract”
• Trans-NanoSim workflow overview section in “Methods”
• Availability of supporting source code and requirements section

2. For the same reason, it could be helpful to add the possibility to simulate reads without transcript abundance file. Instead of real abundances, one can use some approximation (e.g. negative binomial distribution).
Response: We thank the reviewer for this suggestion. We agree that the transcript expression levels change between different experiments, and users may want to test different scenarios. We note that, Trans-NanoSim is quite flexible in this regard; it allows users to provide their own expression profile in a tab-delimited format. If users would like to replace empirical abundance levels with theoretical models, they may do so by generating their own tab-delimited values. We clarified this point in the “Transcript abundance quantification and simulation” paragraph.
3. Quality of plots used in the manuscript can be improved, preferably to vector quality.
Response: Thanks for pointing this out, as effective and good quality figures are indeed important for effective communication. We recreated all figures in vector quality. In this submission, we also attach all figures in the main text as PDF files.

References
1. Laver, Thomas, et al. "Assessing the performance of the oxford nanopore technologies minion." Biomolecular detection and quantification 3 (2015): 1-8.
2. Loman, Nicholas J., Joshua Quick, and Jared T. Simpson. "A complete bacterial genome assembled de novo using only nanopore sequencing data." Nature methods 12.8 (2015): 733-735.

Close
