# Supplementary material for: Trans-NanoSim characterizes and simulates nanopore RNA-sequencing data
Source: Gigascience. 2020 Jun 10;9(6):giaa061. doi: 10.1093/gigascience/giaa061 (PMC7285873; doi:10.1093/gigascience/giaa061)
Supplement: giaa061_Reviewer_1_Report_Original_Submission — Camille Marchet -- 3/10/2020 Reviewed [file giaa061_reviewer_1_report_original_submission.pdf]

Reviewer Report

Title: Trans-NanoSim characterizes and simulates nanopore RNA-seq data

Version: Original Submission **Date:** 3/10/2020

Reviewer name: Camille Marchet

Reviewer Comments to Author:

This article presents a novel method to simulate RNA Oxford Nanopore rads (so-called long reads, along with Pacific BioSciences reads). Read simulation is useful to method developers, and should represent a real typical experiment as close as possible. This contribution is interesting since all other long read simulation tools are meant to reproduce genomic long reads. However, as correctly noted in the document, RNA reads have specific features that require dedicated modeling.
In particular, gene expression and the appearance of RNA minor/major isoforms are important characteristics of a (eukaryotic) RNA sample, since the long reads are likely to span full molecules (or substantial parts of them).
The presented method, Trans-Nanosim, is implemented in a piece of software and builds upon a previous simulator, Nanosim. However, it brings new and essential additions to the previous works. Oxford Nanopore is not a stable technology, read error rates and profiles have changed in the last few years, according to which protocol was released. Trans-Nanosim allows to simulate a read dataset by learning from the characteristics of a real dataset, thus, allows to adapt to the precise protocol one wants to test.
I have a few questions/remarks for the authors:
-"the alignment experimental reads against the reference transcriptome and genome" I would like to clarify whether this order is 1/ transcriptome then 2/ genome here?
-A "staircase effect" can be observed because of truncated reads in ONT experiment. I could not see a mention to truncated reads nor to this effect in the article, however I think this can be an important one. Can this be modeled within the length distribution?
-The unaligned bases are not taken into account to estimate the error rate (though the unaligned reads are used to determine the length distribution). This is a (reasonable) choice, which limitations should be clearly discussed.
-"It is observed that the homopolymer lengths on reads follow normal distribution [...]". Do you have other sources you could cite about this?
-I think sometimes the text contains too many repetitions. For instance, " In this work, we introduce the first ONT transcriptome sequence simulator [...]" could be removed as this has already been clearly stated before.

Level of Interest

Please indicate how interesting you found the manuscript: Choose an item.

Quality of Written English

Please indicate the quality of language in the manuscript: Choose an item.

Declaration of Competing Interests

Please complete a declaration of competing interests, considering the following questions:

- Have you in the past five years received reimbursements, fees, funding, or salary from an organisation that may in any way gain or lose financially from the publication of this manuscript, either now or in the future?
- Do you hold any stocks or shares in an organisation that may in any way gain or lose financially from the publication of this manuscript, either now or in the future?
- Do you hold or are you currently applying for any patents relating to the content of the manuscript?
- Have you received reimbursements, fees, funding, or salary from an organization that holds or has applied for patents relating to the content of the manuscript?
- Do you have any other financial competing interests?
- Do you have any non-financial competing interests in relation to this paper?

If you can answer no to all of the above, write 'I declare that I have no competing interests' below. If your reply is yes to any, please give details below.

1. no 2. no 3. no 4. no 5. no

I agree to the open peer review policy of the journal. I understand that my name will be included on my report to the authors and, if the manuscript is accepted for publication, my named report including any attachments I upload will be posted on the website along with the authors' responses. I agree for my report to be made available under an Open Access Creative Commons CC-BY license (http://creativecommons.org/licenses/by/4.0/). I understand that any comments which I do not wish to be included in my named report can be included as confidential comments to the editors, which will not be published.

Choose an item.

To further support our reviewers, we have joined with Publons, where you can gain additional credit to further highlight your hard work (see: https://publons.com/journal/530/gigascience). On publication of this paper, your review will be automatically added to Publons, you can then choose whether or not to claim your Publons credit. I understand this statement.

Yes Choose an item.
