# Supplementary material for: Trans-NanoSim characterizes and simulates nanopore RNA-sequencing data
Source: Gigascience. 2020 Jun 10;9(6):giaa061. doi: 10.1093/gigascience/giaa061 (PMC7285873; doi:10.1093/gigascience/giaa061)
Supplement: giaa061_Reviewer_2_Report_Original_Submission — Andrey D. Prjibelski, M.Sc. -- 3/13/2020 Reviewed [file giaa061_reviewer_2_report_original_submission.pdf]

Reviewer Report

Title: Trans-NanoSim characterizes and simulates nanopore RNA-seq data

Version: Original Submission **Date:** 3/13/2020

Reviewer name: Andrey D. Prjibelski, M.Sc.

Reviewer Comments to Author:

General comments
The paper entitled "Trans-NanoSim characterizes and simulates nanopore RNA-seq data" describes a new tool for simulating long ONT RNA reads. The authors extend their previous tool NanoSim by adding several modifications, such as transcript abundance and intron retention events. Considering rapid growth of interest to long RNA reads, Trans-NanoSim is a valuable instrument for the scientific community.
The tool itself is available on GitHub, well-documented, easy to install and launch, which, indeed, is a positive aspect of this work. I was able to run quantification and simulation pipelines on my data without any problem, and I'm looking forward to using this tool in my work. Overall, the paper is well-structured and easy to follow. Below I summarize a few non-crucial issues, which, in my opinion, may improve the quality of the manuscript and the tool itself.
Major comments
- My main suggestion is to add a comparison against IsoSeqSim (https://github.com/yunhaowang/IsoSeqSim). Although it was released quite some time ago and may not have such functionality as introducing intron retention, it still has main simulation features such as truncating transcripts and introducing errors according to given profiles.
Minor comments
- Would be useful to provide some trained models for 1-2 typical ONT experiments in the package to allow a user to make a quick start.
- For the same reason, it could be helpful to add the possibility to simulate reads without transcript abundance file. Instead of real abundances, one can use some approximation (e.g. negative binomial distribution).
- Quality of plots used in the manuscript can be improved, preferably to vector quality.

Level of Interest

Please indicate how interesting you found the manuscript: Choose an item.

Quality of Written English

Please indicate the quality of language in the manuscript: Choose an item.

Declaration of Competing Interests

Please complete a declaration of competing interests, considering the following questions:

- Have you in the past five years received reimbursements, fees, funding, or salary from an organisation that may in any way gain or lose financially from the publication of this manuscript, either now or in the future?
- Do you hold any stocks or shares in an organisation that may in any way gain or lose financially from the publication of this manuscript, either now or in the future?
- Do you hold or are you currently applying for any patents relating to the content of the manuscript?
- Have you received reimbursements, fees, funding, or salary from an organization that holds or has applied for patents relating to the content of the manuscript?
- Do you have any other financial competing interests?
- Do you have any non-financial competing interests in relation to this paper?

If you can answer no to all of the above, write 'I declare that I have no competing interests' below. If your reply is yes to any, please give details below.

I declare that I have no competing interests

I agree to the open peer review policy of the journal. I understand that my name will be included on my report to the authors and, if the manuscript is accepted for publication, my named report including any attachments I upload will be posted on the website along with the authors' responses. I agree for my report to be made available under an Open Access Creative Commons CC-BY license (http://creativecommons.org/licenses/by/4.0/). I understand that any comments which I do not wish to be included in my named report can be included as confidential comments to the editors, which will not be published.

Choose an item.

To further support our reviewers, we have joined with Publons, where you can gain additional credit to further highlight your hard work (see: https://publons.com/journal/530/gigascience). On publication of this paper, your review will be automatically added to Publons, you can then choose whether or not to claim your Publons credit. I understand this statement.

Yes Choose an item.
